# Effects of acetazolamide combined with remote ischemic preconditioning on risk of acute mountain sickness: a randomized clinical trial

**DOI:** 10.1186/s12916-023-03209-7

**Published:** 2024-01-02

**Authors:** Moqi Liu, Xueqiao Jiao, Rui Li, Jialu Li, Lu Wang, Liyan Wang, Yishu Wang, Chunmei Lv, Dan Huang, Ran Wei, Liming Wang, Xunming Ji, Xiuhai Guo

**Affiliations:** 1 Department of Neurology, Xuanwu Hospital, Capital Medical University, No.45 Changchun Street, Xicheng District, Beijing, 100053 China; 2 Department of Internal Medicine, Beijing Xiaotangshan Hospital, Beijing, 102211 China

**Keywords:** Acute mountain sickness, Acetazolamide, Remote ischemic preconditioning, PDGF

## Abstract

**Background:**

We aimed to determine whether and how the combination of acetazolamide and remote ischemic preconditioning (RIPC) reduced the incidence and severity of acute mountain sickness (AMS).

**Methods:**

This is a prospective, randomized, open-label, blinded endpoint (PROBE) study involving 250 healthy volunteers. Participants were randomized (1:1:1:1:1) to following five groups: Ripc (RIPC twice daily, 6 days), Rapid-Ripc (RIPC four times daily, 3 days), Acetazolamide (twice daily, 2 days), Combined (Acetazolamide plus Rapid-Ripc), and Control group. After interventions, participants entered a normobaric hypoxic chamber (equivalent to 4000 m) and stayed for 6 h. The primary outcomes included the incidence and severity of AMS, and SpO_2_ after hypoxic exposure. Secondary outcomes included systolic and diastolic blood pressure, and heart rate after hypoxic exposure. The mechanisms of the combined regime were investigated through exploratory outcomes, including analysis of venous blood gas, complete blood count, human cytokine antibody array, ELISA validation for PDGF-AB, and detection of *PDGF* gene polymorphisms.

**Results:**

The combination of acetazolamide and RIPC exhibited powerful efficacy in preventing AMS, reducing the incidence of AMS from 26.0 to 6.0% (Combined vs Control: RR 0.23, 95% CI 0.07–0.70, *P* = 0.006), without significantly increasing the incidence of adverse reactions. Combined group also showed the lowest AMS score (0.92 ± 1.10). Mechanistically, acetazolamide induced a mild metabolic acidosis (pH 7.30 ~ 7.31; HCO_3_^−^ 18.1 ~ 20.8 mmol/L) and improved SpO_2_ (89 ~ 91%) following hypoxic exposure. Additionally, thirty differentially expressed proteins (DEPs) related to immune-inflammatory process were identified after hypoxia, among which PDGF-AB was involved. Further validation of PDGF-AB in all individuals showed that both acetazolamide and RIPC downregulated PDGF-AB before hypoxic exposure, suggesting a possible protective mechanism. Furthermore, genetic analyses demonstrated that individuals carrying the *PDGFA* rs2070958 C allele, rs9690350 G allele, or rs1800814 G allele did not display a decrease in PDGF-AB levels after interventions, and were associated with a higher risk of AMS.

**Conclusions:**

The combination of acetazolamide and RIPC exerts a powerful anti-hypoxic effect and represents an innovative and promising strategy for rapid ascent to high altitudes. Acetazolamide improves oxygen saturation. RIPC further aids acetazolamide, which synergistically regulates PDGF-AB, potentially involved in the pathogenesis of AMS.

**Trial registration:**

ClinicalTrials.gov NCT05023941.

**Supplementary Information:**

The online version contains supplementary material available at 10.1186/s12916-023-03209-7.

## Background

Acute mountain sickness (AMS) is the most common type of high-altitude illness, affecting over a quarter of individuals traveling to above 3500 m [1]. Symptoms of AMS typically arise within 4–12 h after reaching high altitude and can range from mild to severe [2]. Hypoxia is the main contributing factor to the pathophysiological processes of AMS, causing hypoxemia, inflammation, raised pulmonary artery pressure and intracranial pressure, etc [2–5].

For those who need to ascend rapidly to high altitudes, acetazolamide is traditionally the most recommended [6]. This carbonic anhydrase inhibitor functions in several ways to prevent altitude sickness, including inducing diuresis and mild metabolic acidosis, modulating the central and peripheral chemoreceptors, and immunoregulating [7–10]. Typically, acetazolamide can decrease the incidence of AMS by 47% (3000–4000 m, 500 mg/day, 3–5 days prior to ascent) [6], but its use is limited due to the 35–50% incidence of side effects, including paresthesia, taste disturbance, polyuria, electrolyte imbalance, and crystalluria [6, 11, 12]. Moreover, the risk of side effects increases significantly with dosage, while the improvement in efficacy is limited. The risk ratio for the incidence of paresthesia and taste disturbance increased 2.1–3.5 times when the dosage of acetazolamide was augmented from 250 to 500 mg/day [13].

In this context, newly developed non-pharmacological approaches, such as remote ischemic preconditioning (RIPC), have emerged to prevent AMS while avoiding the side effects of the drug [14]. RIPC involves applying brief periods of ischemia and reperfusion to the upper arm, and it has been shown to reduce the incidence of AMS [15], improve oxygen saturation, attenuate the increase in pulmonary artery pressures, and ameliorate attentional function in individuals exposed to high altitude [16–18]. Since acetazolamide and RIPC are both effective in preventing AMS, whether the combination of the two can intensify the effectiveness of AMS prevention deserves further investigation.

**Fig. 1:**
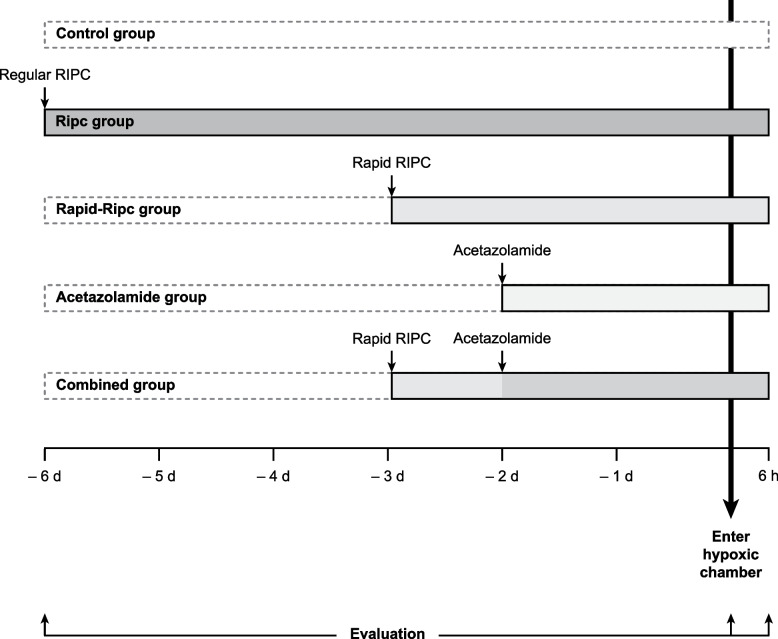
Flowchart describing the study. Participants were randomly assigned to five groups: Control group (no specific intervention), Ripc group (RIPC training twice daily for 6 days), Rapid-Ripc group (RIPC training four times daily for 3 days), Acetazolamide group (125 mg twice daily for 2 days), and Combined group (RIPC training four times daily for 3 days plus acetazolamide 125 mg twice daily for 2 days). After the interventions, participants entered the normobaric hypoxic chamber (approximately equivalent to 4000 m) and stayed for 6 h. Clinical outcomes and laboratory parameters were evaluated at baseline, pre-, and post-hypoxia. RIPC, remote ischemic preconditioning

We conducted a randomized clinical trial to investigate whether the combination of the lowest effective dose of acetazolamide (250 mg/day for 2 days) [12] and a rapid training course of RIPC (four times daily for 3 days) was an effective regimen for minimizing the occurrence and severity of AMS. The study protocol is presented in Fig. 1. We hypothesized that the two methods could act synergistically and their combination could improve clinical outcomes during acute exposure to hypoxia.

## Methods

### Study design and settings

This is a single-center, prospective, randomized, open-label, blinded endpoint (PROBE) study. Participants were randomly assigned to five groups with different interventions: Acetazolamide group, Regular RIPC Training (Ripc) group, Rapid RIPC Training (Rapid-Ripc) group, Acetazolamide plus Rapid RIPC Training (Combined) group, and Control group. After the interventions, participants were exposed to a normobaric hypoxic chamber for 6 h (12% oxygen/88% nitrogen, approximately equivalent to an altitude of 4000 m after adjusting the difference between the actual altitude and the stimulated altitude in a normobaric hypoxic chamber [19]). The primary and secondary outcomes were assessed at baseline, before and after the 6 h of hypoxic exposure. We conducted the study in five rounds due to the large number of participants. Each round lasted for 1 week with 50 participants, with an overall study lasting from Dec. 2021 to Aug. 2022.

### Participants

We recruited healthy lowlander volunteers aged 18 to 50 years without a prior history of high-altitude exposure (> 1500 m) within a month. The exclusion criteria included the following: (1) having any acute or chronic physical or mental disease (including hypertension, diabetes mellitus, coronary heart disease, chronic obstructive pulmonary disease, cerebrovascular disease, migraine, anxiety disorder, depression, and insomnia), (2) peripheral oxygen saturation (SpO_2_) < 95%; (3) having any contradiction for RIPC (including severe soft tissue injury, fracture, or peripheral vascular disease in the upper limbs), (4) allergy to sulfonamides, (5) previous laboratory tests suggesting hypokalemia, hyponatremia or hepatic or renal impairment, (6) taking one or more medications regularly daily, (7) having a smoking or heavy drinking habit, (8) being pregnant or breastfeeding (women), (9) being enrolled or having been enrolled in another clinical trial within three months of this clinical trial.

### Sample size

Sample size was calculated based on previous data. The incidence of AMS at 4000 m altitude without any intervention was about 40% [2, 3]. A study on RIPC for the prevention of AMS showed that a 1-week course of RIPC reduced the incidence of AMS at 3650 m by 25%, from 40 to 30% [15]. In addition, systematic reviews showed that acetazolamide could decrease the incidence of AMS by 47% [6], which corresponded to an incidence of 21% at 4000 m altitude. Therefore, theoretically, the incidence of AMS after using the combination of the two methods was about 16%. With a 5% significance level and 80% power, the sample size of this study included 50 participants in each group, a total of 250 participants.

### Randomization and allocation

Participants were randomized in a 1:1:1:1:1 ratio via simple random sampling. Each participant drew a small ball from a box that contained identically shaped, sized, and colored balls, which were evenly mixed. Each ball was labeled with a number ranging from 001 to 250. Participants with end number zero or five were assigned to Control group, one or six to Combined group, two or seven to Ripc group, three or eight to Rapid-Ripc group, and four or nine to Acetazolamide group.

### Procedures

At baseline, a comprehensive clinical assessment, including consultation and physical examination, was performed for each participant by physicians at Xuanwu Hospital, Capital Medical University in Beijing, China (40 m above sea level). Participants were then randomly assigned to five groups to receive different interventions. The RIPC training was induced by Renqiao Remote Ischemic Conditioning Device. Each training comprised five cycles of bilateral upper limb ischemia and reperfusion, which was induced by two cuffs placed around the upper arms respectively and inflated to a pressure of 180 mmHg for 5 min followed by 5 min of reperfusion by cuff deflation. The protocol was as follows:

1. Ripc group: Started RIPC training twice daily (8 am–4 pm), 6 days before entering the hypoxic chamber. The participants totally finished thirteen times of training.
2. Rapid-Ripc group: Started RIPC training four times daily (8 am–12 pm–4 pm–8 pm), 3 days before entering the hypoxic chamber. The participants totally finished thirteen times of training.
3. Acetazolamide group: Started orally taking acetazolamide 125 mg twice daily (8 am–4 pm), 2 days before entering the hypoxic chamber. The participants totally took the drug five times.
4. Combined group: Participants received protocols of both Acetazolamide group and Rapid-Ripc group. Started orally taking acetazolamide 125 mg twice daily (8 am–4 pm), 2 days prior, and started RIPC training four times daily (8 am–12 pm–4 pm–8 pm), 3 days prior. The participants totally took the drug five times and finished thirteen times of training.

Participants assigned to Control group did not receive specific intervention before entering the hypoxic chamber.

The intervention process was conducted at Beijing Highland Conditioning Medical Center with a dedicated supervision team to ensure completion of the intervention. All participants taking acetazolamide were required to visit the site at 8 am and 4 pm to take their medication, under the supervision of the research physician responsible for dispensing and recording. Participants for the twice-daily RIPC training also came to the site for morning and afternoon training sessions. For the four-times-daily RIPC training, three sessions were conducted on-site, with participants bringing the device home for the final session and providing training photos to the research physician. These photos included photos of the training device screen for each round of inflation-deflation, photos of upper extremities engorgement at the end of the session, and photos of the training environment. The research physician verified and recorded participant’s compliance with the trial protocol. All participants completed the intervention for the trial as required.

After the interventions, participants entered the hypoxic chamber at Beijing Highland Conditioning Medical Center and stayed from 9 am to 3 pm. During the hypoxic exposure, participants stayed seated and performed some low physical activities (e.g., standing, walking, and playing card games). The physician assessed the participants’ vital signs hourly (including blood pressure, heart rate, and SpO_2_). All the participants were encouraged to persist to the end of the 6 h of hypoxia. If severe intolerable symptoms did occur, or if vital signs became exceedingly abnormal (any of the following: SpO_2_ < 70% [20, 21], systolic blood pressure > 160 or < 90 mmHg, diastolic blood pressure > 100 or < 60 mmHg, heart rate > 75% × (220-age) or < 60 beats per minute, respiratory rate > 30 or < 12 breaths per minute), the participant exited the hypoxic room and received oxygen therapy.

### Outcomes

The primary outcomes included the incidence and severity of AMS, and SpO_2_ after hypoxic exposure. The incidence of AMS was evaluated by the 2018 Lake Louise Acute Mountain Sickness Score at the end of the 6 h of hypoxic exposure. A positive AMS was defined as a total score of the four symptoms (headache, gastrointestinal symptoms, fatigue and/or weakness, and dizziness/light-headedness) of at least three points with a headache score of at least one point. The severity of AMS was evaluated by the 2018 Lake Louise Acute Mountain Sickness Score, Acute Mountain Sickness-Cerebral score and Chinese Acute Mountain Sickness score. The participants completed the questionnaires independently. SpO_2_ was measured by finger pulse oximetry (Yuyue Medical Equipment Co., Ltd, Shanghai, Beijing) after hypoxic exposure. Secondary outcomes included systolic and diastolic blood pressure and heart rate after hypoxic exposure. Exploratory outcomes included the hematological parameters evaluated at baseline, pre-hypoxia and post-hypoxia, including venous blood gas, complete blood count (post-hypoxia), human cytokine antibody array evaluation, ELISA validation, and genetic polymorphisms analysis, to explore the mechanisms of the combined regimen. Adverse reactions of acetazolamide or RIPC were also assessed. All data and analyses for the primary and secondary outcomes were assessed by investigators blinded to treatment allocation. A blinded-to-treatment adjudicating group reviewed the data to determine which participants had reached study outcomes and to evaluate safety.

### Sampling collection and analyses

Venous blood samples were drawn at baseline, pre-, and post-hypoxia. During each blood collection, 2 ml venous samples were collected by heparin-coated syringe and analyzed immediately using Vitagas 8E (Kangli Biomedical Inc., Shenzhen, China); 5 ml was collected by Ethylenediamine Tetraacetic Acid (EDTA)-coated vacutainers, and centrifuged at 3000 rpm for 15 min. The plasma was stored at -  80 °C. At post-hypoxia, an additional vacutainer of blood was drawn from all of the participants for a complete blood count test.

During the first round of the study, twenty-four participants were randomly selected from the Control, Acetazolamide, Rapid-Ripc, and Combined group (*n* = 6 for each group) and their samples at baseline, pre-, and post-hypoxia were analyzed for 440 cytokines using the human cytokine antibody array (RayBiotech Inc., Norcross, GA, USA) as described in the manufacturer’s protocol. The 440 cytokines primarily encompass inflammation, angiogenesis, cell migration, cell proliferation, autophagy, apoptosis, oxidative stress, atherosclerosis, cancer-related pathways, etc. The raw fluorescence signal data obtained from the experiments were normalized, compared between groups using moderated t-statistics (data were processed in R/Bioconductor). Differentially expressed proteins (DEPs) were identified based on a fold change > 2 or < 0.5, and *P* value < 0.05. Protein-protein association network was investigated by STRING analysis (http://string-db.org). Based on the literature review, protein signal intensity, and kit accessibility, PDGF-AB was selected and quantitatively validated using ELISA kits (RayBiotech Inc., Norcross, GA, USA) in all the participants, following the manufacturer’s protocol.

### Genetic analyses

Gene polymorphisms of *PDGFA* and *PDGFB* were detected in all subjects. We searched for potentially regulatory single-nucleotide polymorphisms (SNPs) based on previous researches and National Center for Biotechnology Information (NCBI) SNP database (http://www.ncbi.nlm.nih.gov/projects/SNP/). Selected SNPs were required to have a minor allele frequency (MAF) > 0.1 and have been previously studied or reported to be correlated with PDGF protein concentrations. For *PDGFA*, we selected rs1800814 [22], rs2070958 [22], rs9690350 [23], and rs62433334 [22, 24] in introns. For *PDGFB*, we selected rs1800817 [25–27], rs2285099 [28], and rs2285094 [29] in introns, and rs1800818 [25–27, 30] at 5′-UTR.

Genomic DNA was extracted from whole blood following the instructions provided with the Finemag Blood DNA kit (Genfine Biotech Co. Ltd., Beijing, China). The extracted genomic DNA samples were stored at - 20 °C until analysis. The polymerase chain reaction (PCR) primers are listed in Additional file 1: Table S1. Following PCR amplification and purification, the DNA sequences were analyzed using the ABI 3730xl DNA Analyzer. The assays were repeated for 5% of the samples, and the results were 100% concordant. Investigators involved in SNP analyses were blinded to participants’ clinical data.

### Statistical analyses

Continuous variables were expressed as means with standard deviation (SD). Normality of distribution for continuous variables was assessed using the Shapiro-Wilk test. Categorical variables were expressed as counts and percentages. For the primary outcomes, the planned comparison of the incidence of AMS between groups was conducted using *χ*^2^ test. The risk ratio with confidence intervals was reported. The AMS score between groups were investigated using one-way ANOVA or Kruskal-Wallis test. The comparisons of SpO_2_ after hypoxic exposure were conducted using paired two-way ANOVA (different time point after hypoxia vs different intervention groups). For secondary outcomes, the systolic and diastolic blood pressure and heart rate after hypoxic exposure were investigated using paired two-way ANOVA. For exploratory outcomes, we pre-specified the comparisons of venous blood gas parameters after interventions using paired two-way ANOVA. We pre-specified the cytokine antibody research in each of Control, Rapid-Ripc, Acetazolamide, and Combined group before and after hypoxia. After identifying PDGF-AB, PDGF-AB levels were validated and investigated using paired two-way ANOVA, and means with SD and mean differences with their 2-sided 95% confidence interval (CI) were reported. We further divided the participants into subgroups based on AMS (+)/(-) and based on different intervention groups to analyze the change of PDGF-AB. Analyses were conducted with GraphPad Prism version 9.0 (GraphPad Software: San Diego, CA, USA). Turkey’s multiple comparisons were performed between each pair of groups.

Genetic data of *PDGF* were tested for Hardy–Weinberg equilibrium using *χ*^2^-test. Genotypes were analyzed in codominant, dominant, recessive, and log-additive model using SNPStats online software (http://bioinfo.iconcologia.net/SNPstats). Allele frequencies and genotype distribution were compared between AMS (+) and AMS (-) groups using the *χ*^2^-test. Binary logistic regression was used to analyze associations between genotypes and AMS, which were further adjusted for age, sex, and body mass index (BMI). We divided the participants into subgroups based on their genotype and interventions, and analyzed the PDGF-AB levels in different subgroups. The level of significance was set as a 2-sided *P* value less than 0.05.

### Study approval

The study was approved by the Ethics Committee of the Xuanwu Hospital, Capital Medical University and registered on ClinicalTrials.gov (NCT05023941). All participants provided written informed consent. The study was conducted in compliance with the Declaration of Helsinki and Good Clinical Practice guidelines.

## Results

### Participant characteristics

**Fig. 2:**
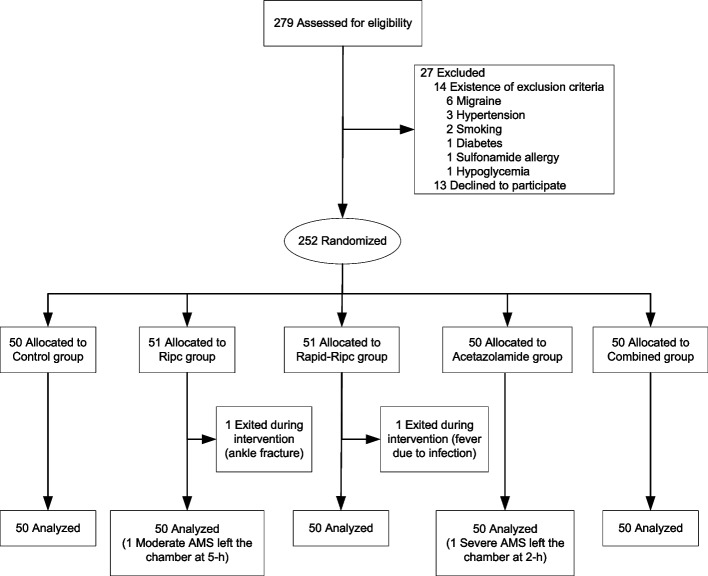
Study profile

From 279 subjects who signed up and met the enrollment criteria, 252 were randomized to different groups. Two terminated the study prematurely during the intervention phase. Thus, a total of 250 participants finally entered the hypoxic chamber and were included in the analysis, among them two left the chamber at 2-h or 5-h hypoxic exposure due to severe discomfort (Fig. 2). The participants consisted of 152 (60.8%) women and 98 (39.2%) men, with a mean age of 30.38 ± 7.17 years. The baseline characteristics are presented in Additional file 1: Table S2.

### Primary outcomes

**Fig. 3:**
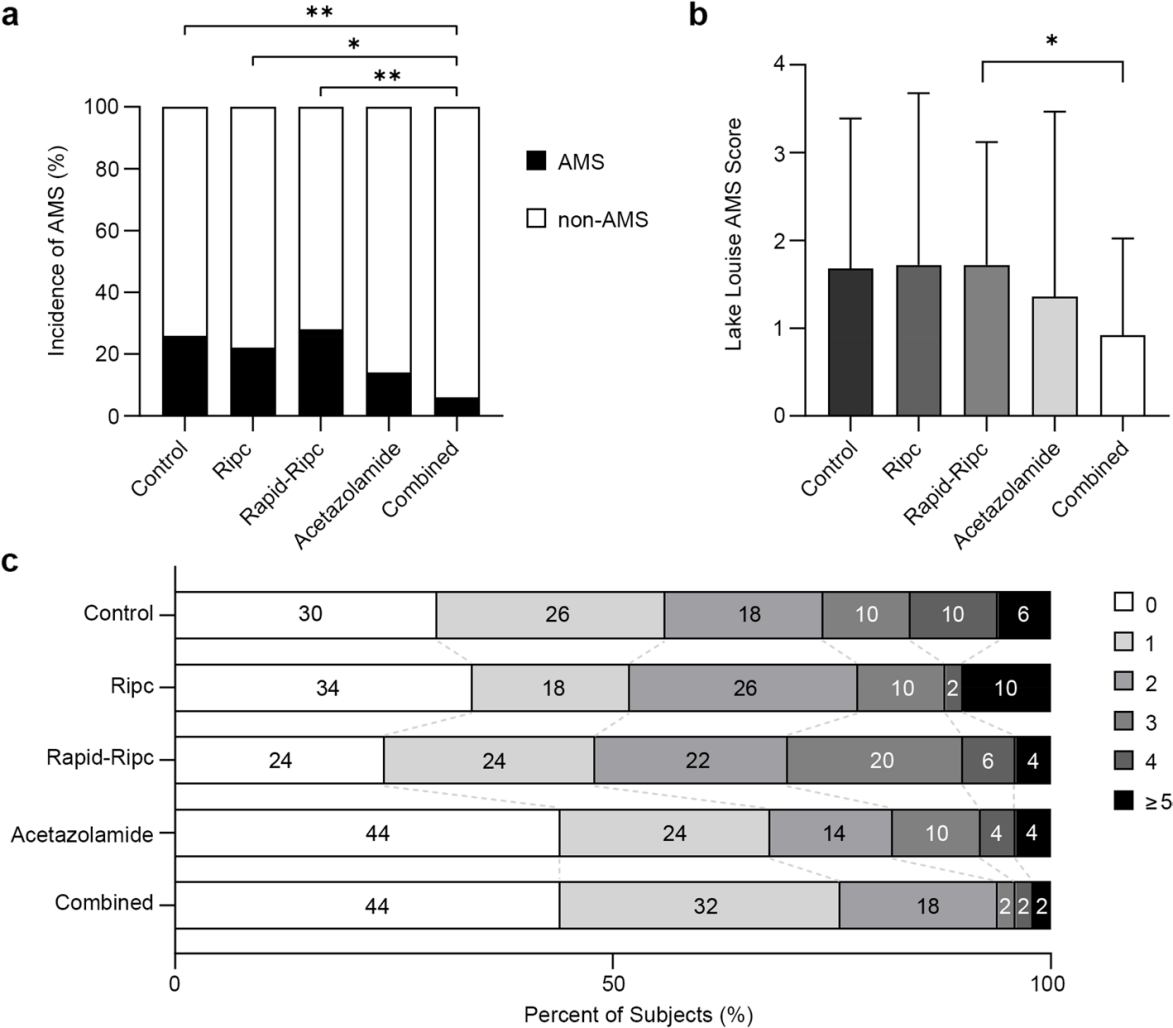
Incidence and severity of AMS assessed using the 2018 Lake Louise Acute Mountain Sickness Score at 6 h of hypoxic exposure in different groups. **a** AMS incidence rates. **b** Lake Louise AMS Scores. Data presented as mean with SD. **c** Distribution of AMS Scores. The numbers within the bars represent the percentage of subjects with each score

A total of 48 (19.2%) participants developed AMS in our study. Combined group exhibited the lowest incidence and mildest severity of AMS. The number of AMS-positive participants in Control, Ripc, Rapid-Ripc, Acetazolamide, and Combined group were 13 (26%), 11 (22%), 14 (28%), 7 (14%), and 3 (6%), respectively (*P* = 0.030, Fig. 3a). Statistical differences were found between Combined and Control (RR 0.23, 95% CI 0.07 to 0.70, *P* = 0.006), Ripc (RR 0.27, 95% CI 0.09 to 0.84, *P* = 0.021), and Rapid-Ripc group (RR 0.21, 95% CI 0.07 to 0.64, *P* = 0.003). No significant difference was observed between Acetazolamide and the other groups (vs Control, *P* = 0.134; vs Ripc, *P* = 0.298; vs Rapid-Ripc, *P* = 0.086; vs Combined, *P* = 0.183).

The Lake Louise AMS Score of the five groups after hypoxic exposure were 1.68 ± 1.71, 1.72 ± 1.96, 1.72 ± 1.40, 1.36 ± 2.11, and 0.92 ± 1.10, respectively (Fig. 3b). Combined group exhibited lower scores than the other groups, and the difference was statistically significant when compared to Rapid-Ripc group (*P* = 0.034). The AMS-Cerebral Score and the Chinese AMS score also showed that Combined group was less symptomatic, but there were no significant differences between the groups (Additional file 1: Figure S1). We further analyzed the distribution of AMS scores in five groups (Fig. 3c). Fewer participants in Acetazolamide and Combined groups developed symptoms (defined as AMS score ≥ 1) and eventually developed AMS. The proportion of subjects scoring three or higher was less in Combined group than in Acetazolamide group (6% vs 18%, *P* = 0.065). Regarding AMS symptoms, 107 (42.8%) participants experienced headaches, followed by 101 (40.4%) developed dizziness/light-headedness, 88 (35.2%) fatigue and/or weakness, and 26 (10.4%) gastrointestinal symptoms. Fewer participants in Acetazolamide and Combined groups suffered from headaches (Table 1).

**Table 1:** Distribution of AMS symptoms at 6 h of hypoxic exposure in different groups

	Groups					
AMS symptoms, No. (%)	Control group	Ripc group	Rapid-Ripc group	Acetazolamide group	Combined group	*P* value
Headache	24 (48.0)	22 (44.0)	29 (58.0)	16 (32.0)	16 (32.0)	0.041
Gastrointestinal symptoms	5 (10.0)	6 (12.0)	6 (12.0)	5 (10.0)	4 (8.0)	0.963
Fatigue and/or weakness	19 (38.0)	21 (42.0)	12 (24.0)	14 (28.0)	12 (24.0)	0.168
Dizziness/light-headedness	25 (50.0)	21 (42.0)	23 (46.0)	20 (40.0)	12 (24.0)	0.084

**Fig. 4:**
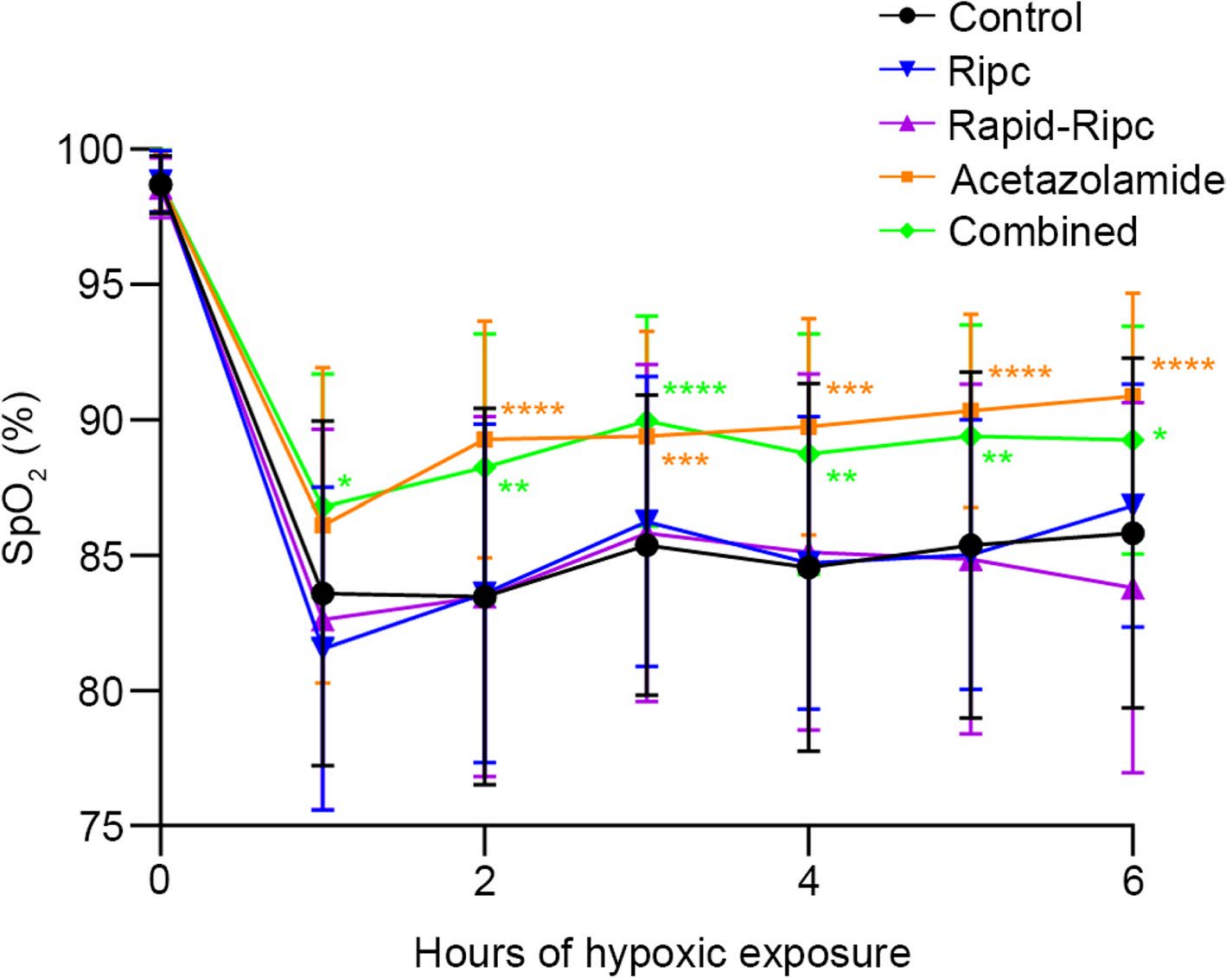
Change of SpO_2_ in different groups during the 6 h of hypoxic exposure. After hypoxic exposure, SpO_2_ reached its minimum at 1-h hypoxia. At 6-h hypoxia, Acetazolamide and Combined group showed significantly higher SpO_2_ than the other groups. Data are mean with SD. ^*^P < 0.05, ^**^P < 0.01, ^***^P < 0.001, ^****^P < 0.0001 vs. Control group

SpO_2_ was assessed in five groups after 6 h of hypoxic exposure (Fig. 4). Combined and Acetazolamide groups showed significantly ameliorated SpO_2_ compared to the other groups, and the differences were detected as early as 1 h after being exposed to hypoxia. At 6 h, the SpO_2_ in Control, Ripc, Rapid-Ripc, Acetazolamide, and Combined group were 85.82 ± 6.40%, 86.90 ± 4.46%, 83.80 ± 6.77%, 90.92 ± 3.77%, and 89.24 ± 4.17%, respectively. Significant differences were found between Combined and Acetazolamide with other groups (Acetazolamide vs Control: 5.06%, 95% CI 2.12 to 8.00, *P* < 0.001; vs Ripc: 4.04%, 95% CI 1.07 to 7.01, *P* < 0.001; vs Rapid-Ripc: 7.08%, 95% CI 4.13 to 10.02, *P* < 0.001; Combined vs Control: 3.42%, 95% CI 0.49 to 6.35, *P* = 0.020; vs Rapid-Ripc: 5.44%, 95% CI 2.51 to 8.37, *P* < 0.001).

### Safety and adverse events

Drug-related adverse reactions occurred in 44 (44.0%) participants taking acetazolamide, with paresthesia being the most commonly reported symptom, followed by polyuria and taste disturbance (Table 2). The majority of these reactions were classified as mild. There was no significant difference between Acetazolamide and Combined group.

**Table 2:** Drug-related adverse reactions

Symptoms (No. (%))	Acetazolamide group	Combined group	*P* value
Total	25 (50.0)	19 (38.0)	0.227
Paresthesia	18 (36.0)	15 (30.0)	0.523
Polyuria	8 (16.0)	7 (14.0)	0.779
Taste disturbance	1 (2.0)	1 (2.0)	1.000
Headache	4 (8.0)	0	0.117
Rash	1 (2.0)	2 (4.0)	0.558
Nausea	1 (2.0)	0	0.315

RIPC-related adverse reaction, mainly bleeding spots on the skin of the upper arm, occurred in 45 (30%) participants, including 11 (22%), 20 (40%), and 14 (28%) in Ripc, Rapid-Ripc, and Combined group, respectively (*P* = 0.135). Increasing the intensity of RIPC training did not result in a significant increase in the incidence of bleeding spots on the upper arm.

The total number of adverse reactions in Ripc, Rapid-Ripc, Acetazolamide, and Combined group were 11 (22%), 20 (40%), 25 (50%), and 27 (54%), respectively. The incidence in Ripc group was lower than the other groups, and the differences were significant compared to Acetazolamide (*P* = 0.004) or Combined group (*P* = 0.001). The incidence of adverse effects did not significantly increase when Acetazolamide and Rapid-Ripc were used in combination (Combined group) compared to when Acetazolamide or Rapid-Ripc was used alone (*P* = 0.689 and *P* = 0.161, respectively). Taken together, Combined group had no significant increase in the incidence of adverse reactions.

### Secondary outcomes

The systolic and diastolic blood pressure and heart rate showed little fluctuation during the hypoxic exposure and did not exhibit significant difference between the groups. The results are showed in Additional file 1: Figure S2.

### Exploratory outcomes

#### Venous blood gas analysis for acid-base evaluation

Given the effectiveness of Combined group, we further explored the synergistic mechanisms underlying the two methods. Acetazolamide and Combined groups showed significantly lower pH, actual bicarbonate, standard bicarbonate, and base excess at pre-hypoxia, indicating a mild metabolic acidosis induced by the intervention (Additional file 1: Table S3). The P50 was higher in Acetazolamide and Combined groups than in the other groups at pre-hypoxia, implying a right shift of the oxygen dissociation curve. Since the parameters in Combined group did not change significantly compared to Acetazolamide group, the effect of regulating acid-base balance was mainly from acetazolamide. Taken together the results of venous blood gas and SpO_2_, we suggested that acetazolamide induced mild metabolic acidosis and increased SpO_2_ levels, exerting its anti-hypoxic effect.

#### Inflammation and cytokine antibody array evaluation during hypoxia

Since Rapid-Ripc did not demonstrate a significant ability to increase oxygen saturation, we proposed that it might enhance the anti-AMS effect through an anti-inflammatory pathway. To test this hypothesis, we firstly compared the inflammatory response between individuals with AMS (+) and AMS (-) after exposure to hypoxia. Our findings revealed that AMS (+) individuals exhibited a more pronounced inflammatory response, characterized by neutrophil-dependent inflammation, compared to AMS (-) individuals (Additional file 1: Table S4).

**Fig. 5:**
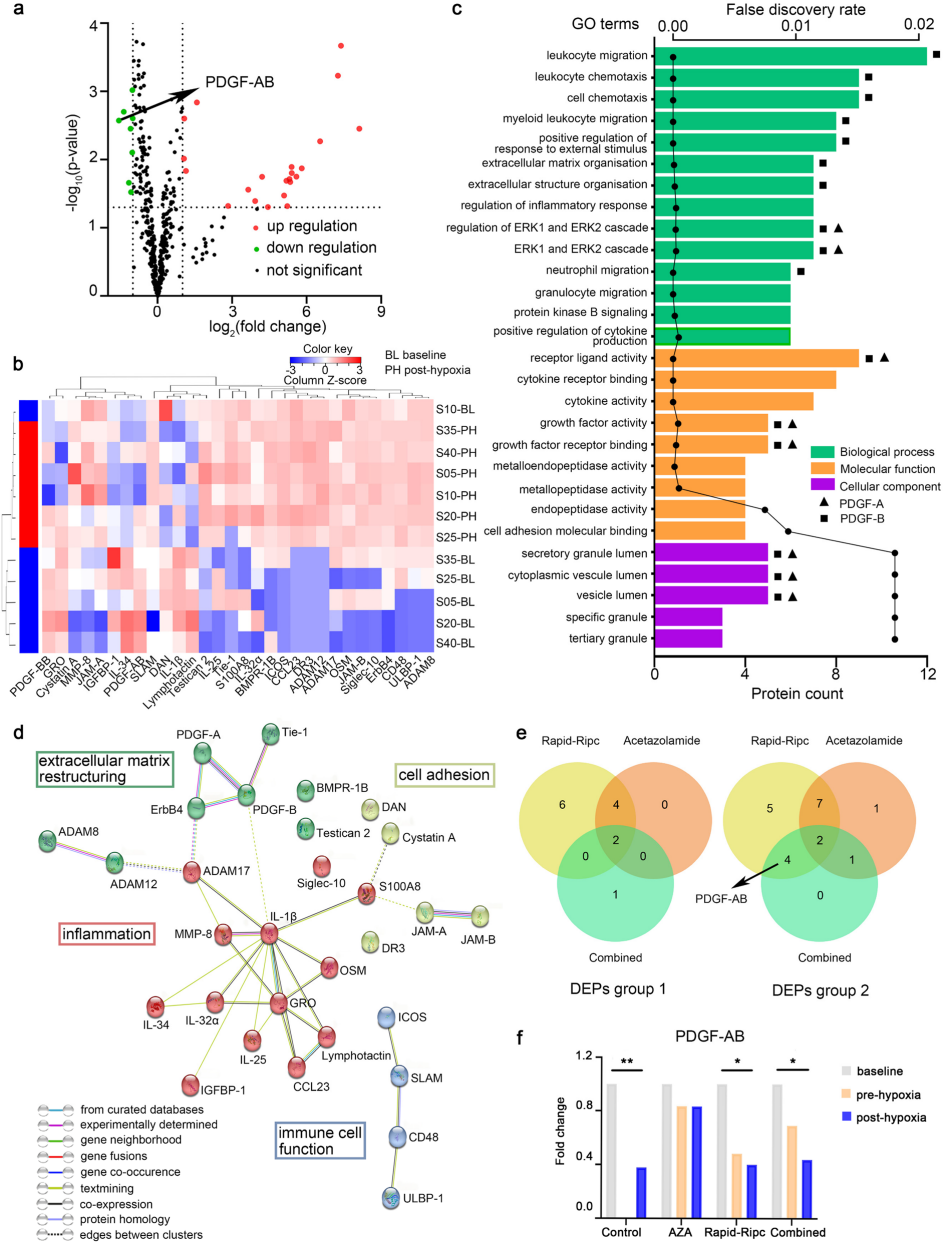
Hypoxia-associated proteins and target protein selection for Combined mechanism. **a** Volcano plot of hypoxia-associated proteins. Hypoxia-associated proteins refer to DEPs in Control group subjects (*n* = 6) between baseline and post-hypoxia. The proteins are showed according to their log_2_ (fold change) (*X*-axis) and significance (*Y*-axis: − log_10_ *p* value). Red dots represent proteins with a significant fold change > 2; green dots, proteins with a significant fold change < 0.5; black dots, proteins with no significant change. A total of 30 hypoxia-associated proteins were identified, among which 22 were upregulated and 8 were downregulated. **b** Heatmap of expression profiling of hypoxia-associated proteins. Each line represents a sample, each column is a protein, and the colors represent different expression levels. **c** Gene Ontology (GO) term enrichment analysis of hypoxia-associated proteins. *X*-axis indicates the number of proteins; *Y*-axis represents the different GO terms. **d** Protein–protein association network of hypoxia-associated proteins by STRING analysis. The network nodes are proteins, which were clustered to four clusters using kmeans clustering. The proteins in each cluster contributed to a common function: red nodes for inflammation, green for extracellular matrix restructuring, yellow for cell adhesion, and blue for immune cell function. Differently colored lines represent different types of evidence used in predicting the associations. **e** Venn diagrams of hypoxia-associated proteins between baseline and pre-hypoxia (DEPs group 1) and between baseline and post-hypoxia (DEPs group 2) in each of the Acetazolamide (*n* = 6), Rapid-Ripc (*n* = 6), and Combined group (*n* = 6). A total of fifteen proteins in the overlapping region were focused. After reviewing the literature and considering the fluorescence signal intensity, PDGF-AB was selected for further validation. **f** Expression of PDGF-AB. *X*-axis represents different groups; *Y*-axis represents fold change. *N* = 6 in each group. AZA, acetazolamide. *P < 0.05, **P < 0.01

Secondly, we identified the hypoxia-associated proteins using a 440-humain cytokine antibody array. We analyzed protein changes between baseline and post-hypoxia in Control group (*n* = 6). A total of 30 DEPs were identified, among which 22 were upregulated and 8 were downregulated (Fig. 5a, b, and Additional file 1: Table S5). Analysis of Gene Ontology (GO) showed that these hypoxia-associated proteins were mainly related to immune-inflammatory processes, characterized by leukocyte migration, chemotaxis cytokine activity, etc., and were mostly secreted or located in the cytoplasm (Fig. 5c). The protein-protein association network further implied that the hypoxia-associated proteins were involved in inflammation, restructuring of the extracellular matrix, cell adhesion, and immune function (Fig. 5d).

Thirdly, we examined the impact of different interventions on hypoxia-associated proteins and focused on potential targets of Combined mechanism. We analyzed the changes of hypoxia-associated proteins in each of the Acetazolamide (*n* = 6), Rapid-Ripc (*n* = 6), and Combined group (*n* = 6) between baseline and pre-hypoxia (DEPs group 1, representing the effect of intervention) and between baseline and post-hypoxia (DEPs group 2, representing the effect of intervention and/or hypoxia). We focused on the proteins in the overlapping regions in both DEPs group 1 and 2. Thus, a total of 15 proteins constituted candidate proteins for exploring the synergistic mechanism (IGFBP-1, DR3, Lymphotactin, PDGF-AB, GRO, Tie-1, IL-32α, ErbB4, Siglec-10, BMPR-1B, ADAM8, ADAM12, JAM-B, CD48, ULBP-1) (Fig. 5e, and Additional file 1: Tables S6 and S7 for details). After reviewing the literature, considering the fluorescence signal intensity, kit accessibility, and other factors, we selected PDGF-AB (Fig. 5f) for ELISA validation. PDGF-BB was also validated because it belonged to the same family as PDGF-AB and was also a hypoxia-associated protein observed in our study. 

#### ELISA assessment for PDGF-AB in all participants

**Fig. 6:**
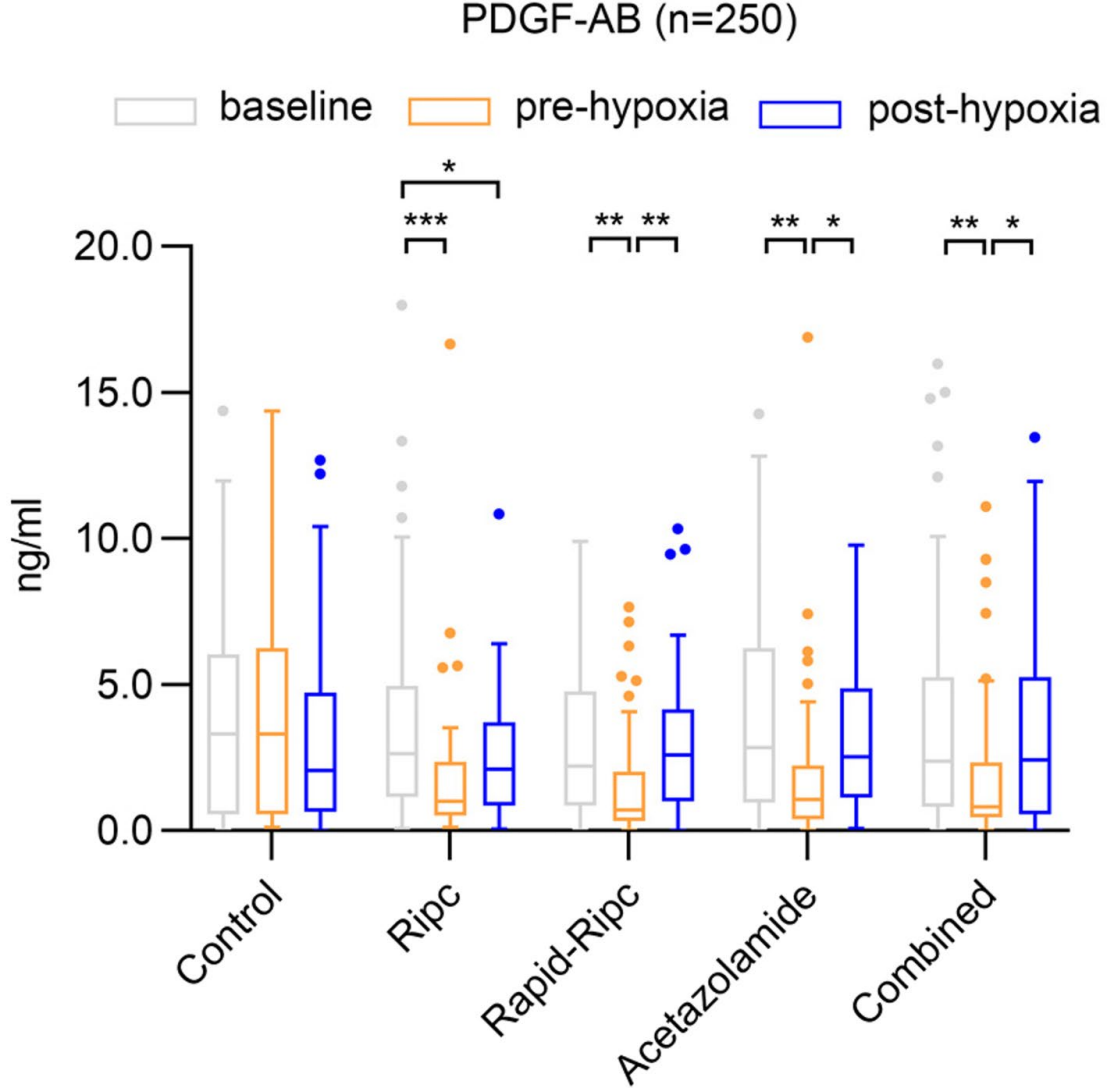
PDGF-AB levels in all subjects validated by ELISA. PDGF-AB levels at baseline, pre-, and post-hypoxia in each group are presented. All intervention groups exhibited a decrease in PDGF-AB levels from baseline to pre-hypoxia, followed by an increase in levels after hypoxic exposure. The pre-hypoxic PDGF-AB levels in Ripc (*P* = 0.014), Rapid-Ripc (*P* < 0.001), Acetazolamide (*P* = 0.018), and Combined groups (*P* = 0.010) were significantly lower than those in Control group. Data are presented in box-whisker plots using Tukey method: the box extends from the 25th to 75th percentiles, and the line in the middle of the box represents the median. The upper whisker refers to the 75th percentile plus 1.5 times inter-quartile distance (IQD), and the lower whisker refers to the 25th percentile minus 1.5 IQD. Outliers are plotted individually. Statistical analyses included all the individual values. ^*^P < 0.05, ^**^P < 0.01, ^***^P < 0.001

The ELISA result of PDGF-AB is shown in Fig. 6. No significant differences were found in PDGF-AB levels between baseline and post-hypoxia, except for Ripc group (*P* = 0.018). However, a dynamic change of PDGF-AB was observed in all intervention groups, with the intervention reducing the level of protein during normoxia and hypoxia increasing it again (baseline to pre-hypoxia, mean difference: Control group:-0.02 ng/ml, 95% CI -0.4 to 0.4, *P* = 0.994; Ripc group:-1.95 ng/ml, 95% CI -3.1 to -0.8, *P* < 0.001; Rapid-Ripc group:-1.57 ng/ml, 95% CI -2.7 to -0.4, *P* = 0.006; Acetazolamide group:-2.06 ng/ml, 95% CI -3.6 to -0.5, *P* = 0.006; Combined group:-2.05 ng/ml, 95% CI -3.4 to -0.7, *P* = 0.002). The pre-hypoxic PDGF-AB levels in Ripc (1.92 ± 2.57 ng/ml, *P* = 0.014 compared to Control), Rapid-Ripc (1.58 ± 1.89 ng/ml,* P* < 0.001), Acetazolamide (1.94 ± 2.71 ng/ml,* P* = 0.018), and Combined groups (1.89 ± 2.47 ng/ml,* P* = 0.010) were significantly lower than those in Control group (3.90 ± 3.41 ng/ml).

To further investigate the relationship between PDGF-AB and AMS, we analyzed PDGF-AB levels at baseline, pre-, and post-hypoxia within the AMS (+) and AMS (-) groups, respectively (Additional file 1: Figure S3). The results showed that in AMS (-) subjects, there was a significant decrease in PDGF-AB from baseline to pre-hypoxia (-1.75 ng/ml, 95% CI -2.3 to -1.2, *P* < 0.001), while in AMS (+) subjects, no significant reduction was observed (-0.61 ng/ml, 95% CI -1.7 to 0.5, *P* = 0.355). The reduction of PDGF-AB levels was significantly different between the two groups (-1.75 ng/ml vs -0.61 ng/ml, 95%CI -2.24 to -0.03, *P* = 0.043). After excluding Control group to investigate the effect of the intervention, the results were similar with a significant decrease of PDGF-AB in AMS (-) (-2.13 ng/ml, 95% CI -2.8 to -1.4, *P* < 0.001) but not in AMS (+) (-0.87 ng/ml, 95% CI -2.3 to 0.6, *P* = 0.328). Therefore, a significant reduction in PDGF-AB levels before entering hypoxia was associated with the non-occurrence of AMS.

Further analyses of the different intervention groups showed that both acetazolamide, Ripc, Rapid-Ripc reduced PDGF-AB in AMS (-) subjects (Acetazolamide group:-2.29 ng/ml, 95% CI -4.0 to -0.6, *P* = 0.006; Ripc group:-2.31 ng/ml, 95% CI -3.6 to -1.0, *P* < 0.001; Rapid-Ripc group:-1.72 ng/ml, 95% CI -3.0 to -0.5, *P* = 0.004), but not in AMS (+) (Acetazolamide group:-0.60 ng/ml, 95% CI -4.5 to 3.3, *P* = 0.887; Ripc group:-0.68 ng/ml, 95% CI -3.1 to 1.7, *P* = 0.724; Rapid-Ripc group:-1.18 ng/ml, 95% CI -4.3 to 2.0, *P* = 0.593) (Additional file 1: Figure S4). After the combination of acetazolamide and Rapid-Ripc, the reduction of PDGF-AB remained significant in AMS (-) subjects (-2.14 ng/ml, 95% CI -3.6 to -0.7, *P* = 0.002). Taken together with previous results, both acetazolamide and RIPC were able to reduce PDGF-AB before hypoxic exposure, and individuals whose PDGF-AB was reduced by acetazolamide and/or RIPC did not develop AMS. The results for PDGF-BB are showed in Additional file 1: Figures S5-S7.

#### Detection of *PDGFA* gene polymorphisms

Given the close association of PDGF-AB with AMS and the effects of interventions, we further explored additional genetic evidence to support this relationship. A total of 233 subjects (93.2%) were successfully genotyped in our study. Genotype frequencies of each of the analyzed SNPs were compatible with the Hardy–Weinberg equilibrium (*P* > 0.050). Among the eight SNPs tested, three SNPs (*PDGFA*-rs2070958, *PDGFA*-rs9690350, and *PDGFA*-rs1800814) were found to be associated with the risk of developing AMS, even after adjusted by age, sex, and BMI. No significant association was observed between the selected *PDGFB* SNPs and AMS. The negative results of the other five SNPs are shown in Additional file 1: Table S8.

The distributions of the *PDGFA*-rs2070958 C/T alleles and *PDGFA*-rs9690350 G/C alleles were significantly different between AMS (+) and AMS (-) groups, with AMS (-) subjects exhibited a higher frequency of the T in rs2070958 and the C in rs9690350 (*P* < 0.05). AMS (-) subjects also carried more A than G alleles in *PDGFA*-rs1800814, although the observed difference was marginally significant (*P* = 0.053). Under the dominant model, the dominant genotypes of the three SNPs were associated with significantly increased AMS risk (OR > 1; *P* < 0.05) after adjusting for age, sex, and BMI. Additionally, under the log-additive model, these three SNPs were all associated with an increased risk of AMS (OR > 1; *P* < 0.05) (Table 3).

**Table 3:** Associations analyses between SNPs and AMS

Model	Allele/Genotype	AMS (+) *n* = 42	AMS (−) *n* = 191	OR (95% CI)	*P* value	OR (95% CI)^a^	*P* value^a^
**rs2070958 C > T**							
Allele	C	52 (61.9%)	189 (49.5%)	-	0.039	-	-
	T	32 (38.1%)	193 (50.5%)	-		-	
Codominant	CC	16 (38.1%)	42 (22.0%)	1	0.086	1	0.07
	CT	20 (47.6%)	105 (55.0%)	2.00 (0.95–4.23)		2.21 (1.03–4.76)	
	TT	6 (14.3%)	44 (23.0%)	2.79 (1.00–7.82)		2.81 (0.99–8.02)	
Dominant	CC	16 (38.1%)	42 (22.0%)	1	0.035	1	0.024
	CT/TT	26 (61.9%)	149 (78.0%)	2.18 (1.07–4.44)		2.35 (1.13–4.87)	
Recessive	CC/CT	36 (85.7%)	147 (77.0%)	1	0.19	1	0.26
	TT	6 (14.3%)	44 (23.0%)	1.80 (0.71–4.54)		1.69 (0.66–4.33)	
Log-additive	-	-	-	1.74 (1.04–2.89)	0.031	1.78 (1.05–3.02)	0.029
**rs9690350 G > C**							
Allele	G	59 (70.2%)	221 (57.6%)	-	0.032	-	-
	C	25 (29.8%)	163 (42.4%)	-		-	
Codominant	GG	21 (50.0%)	63 (32.8%)	1	0.09	1	0.092
	CG	17 (40.5%)	95 (49.5%))	1.86 (0.91–3.81)		1.98 (0.95–4.12)	
	CC	4 (9.5%)	34 (17.7%)	2.83 (0.90–8.93)		2.67 (0.84–8.53)	
Dominant	GG	21 (50.0%)	63 (32.8%)	1	0.038	1	0.033
	CG/CC	21 (50.0%)	129 (67.2%)	2.05 (1.04–4.02)		2.12 (1.06–4.22)	
Recessive	GG/CG	38 (90.5%)	158 (82.3%)	1	0.17	1	0.24
	CC	4 (9.5%)	34 (17.7%)	2.04 (0.68–6.11)		1.87 (0.62–5.67)	
Log-additive	-	-	-	1.75 (1.04–2.92)	0.029	1.75 (1.03–2.98)	0.033
**rs1800814 G > A**							
Allele	G	52 (61.9%)	192 (50.3%)	-	0.053	-	-
	A	32 (38.1%)	190 (49.7%)	-		-	
Codominant	GG	16 (38.1%)	43 (22.5%)	1	0.11	1	0.077
	GA	20 (47.6%)	106 (55.5%)	1.97 (0.93–4.16)		2.22 (1.03–4.78)	
	AA	6 (14.3%)	42 (22.0%)	2.60 (0.93–7.30)		2.69 (0.94–7.69)	
Dominant	GG	16 (38.1%)	43 (22.5%)	1	0.042	1	0.026
	GA/AA	26 (61.9%)	148 (77.5%)	2.12 (1.04–4.31)		2.33 (1.12–4.83)	
Recessive	GG/GA	36 (85.7%)	149 (78.0%)	1	0.25	1	0.31
	AA	6 (14.3)	42 (22.0%)	1.69 (0.67–4.28)		1.61 (0.63–4.13)	
Log-additive	-	-	-	1.69 (1.01–2.82)	0.042	1.75 (1.03–2.98)	0.035

*AMS* acute mountain sickness, *BMI* body mass index, *OR* odds ratio, *CI* confidence interval

^a^Adjusted for age, sex and BMI

**Fig. 7:**
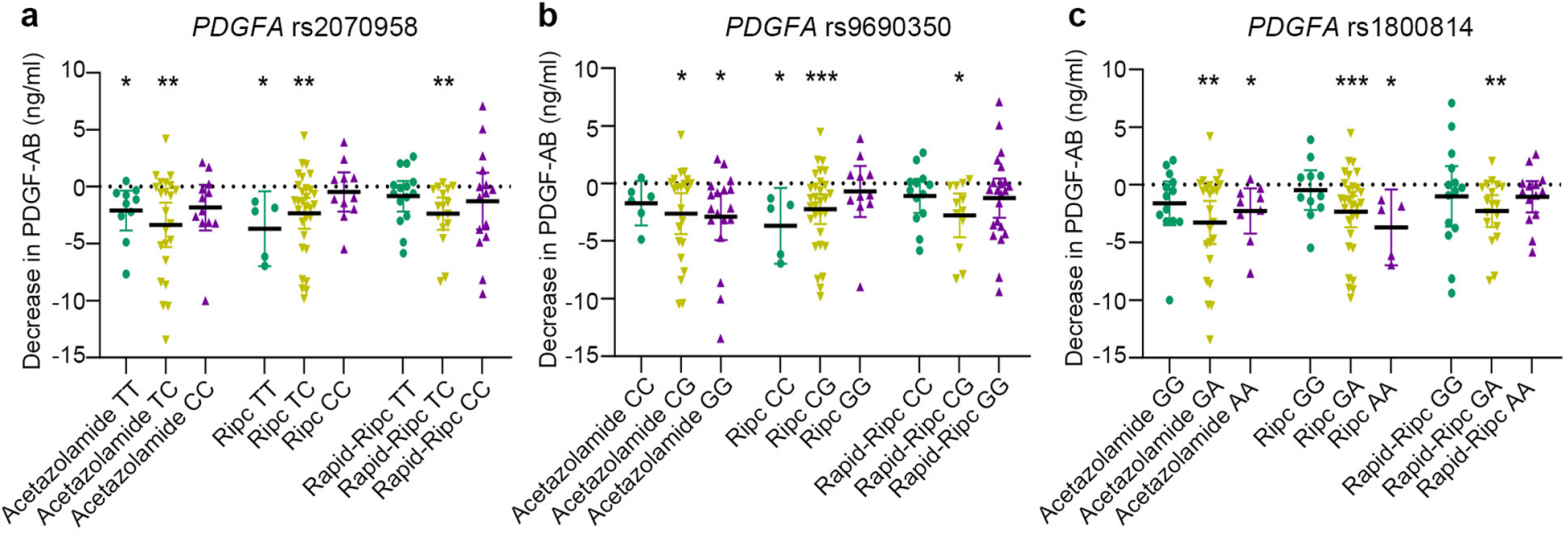
Decrease in PDGF-AB from baseline to pre-hypoxia in different subgroups of *PDGFA* SNPs. Each point in the graph represents the difference in PDGF-AB concentrations from baseline to pre-hypoxia for an individual. The asterisk (*) indicates a significant decrease of PDGF-AB from baseline to pre-hypoxia within the specific genotype group. **a** Decrease in PDGF-AB levels in different subgroups of *PDGFA* rs2070958. Individuals carrying the rs2070958 T allele (TT or TC) in both Acetazolamide and Ripc groups showed a significant decrease in PDGF-AB levels. **b** Decrease in PDGF-AB levels in different subgroups of *PDGFA* rs9690350. Individuals carrying the rs9690350 C allele (CC or CG) in Ripc group showed a significant decrease in PDGF-AB levels. **c** Decrease in PDGF-AB levels in different subgroups of *PDGFA* rs1800814. Individuals carrying the rs1800814 A allele (AA or GA) in both Acetazolamide and Ripc groups showed a significant decrease in PDGF-AB levels. Data are individual values, and mean with SD are showed

To further investigate the associations between *PDGFA* SNPs and various interventions in our study, we conducted an analysis of PDGF-AB concentrations across different genotypes within both acetazolamide and instrument groups (Fig. 7; Additional file 1: Table S9 for details). Individuals carrying rs2070958 T allele or individuals carrying rs1800814 A allele were more likely to respond to acetazolamide or RIPC and showed a significant decrease in PDGF-AB levels with interventions, while individuals carrying rs9690350 C allele genotype were more likely to respond to RIPC. Since *PDGFA* polymorphisms were associated with AMS as well as a decrease in PDGF-AB after interventions, the genetic evidences further demonstrated a relationship between PDGF-AB and AMS.

## Discussion

Our study confirmed a powerful efficacy of combining acetazolamide with RIPC for preventing acute mountain sickness, and explored the synergistic mechanisms of these two methods. The incidence of AMS in Control group was 26%, which was consistent with previous studies that reported a prevalence of 25–43% at 3500–4300 m [1–3]. However, it was still lower than our pre-estimated incidence of 40%, which may be attributed to the limited exposure time, normobaric hypoxia instead of hypobaric hypoxia of a real altitude environment, the low activity levels in the chamber, and the reduced anxiety of participants who chatted and played card games in the chamber [31, 32]. Acetazolamide showed a strong effect with an incidence of 14%, near to the reported rate of 16.9% at 3000–4000 m [6]. Despite the non-significant result of using the instrument alone, the combination of acetazolamide and Rapid-Ripc showed the most favorable protective effect, reducing the incidence of AMS from 26 to 6%. Combined group also exhibited the lowest AMS score compared to other groups while did not result in a significant increase in the incidence of adverse reactions.

To avoid hypoxemia, acetazolamide induces mild metabolic acidosis and improves SpO_2_ after acute hypoxic exposure. Control group showed an average SpO_2_ of 86% at 6 h, in accordance with the range of 80–90% reported in previous studies at 3000–4000 m [20, 21, 33]. The SpO_2_ level of Acetazolamide group significantly increased to 91% after 6 h of hypoxia. Results of peripheral venous blood gas [34, 35] suggested that acetazolamide induced mild metabolic acidosis, which led to hyperventilation and an improvement of the oxygen supply under hypoxic conditions [10, 36]. On the other hand, our study, as well as a previous clinical trial [15], showed that RIPC had a limited role in improving SpO_2_, despite its ability to enhance tissue oxygen exchange in mouse models [37]. Given that the combination of acetazolamide and RIPC performed the most potent clinical effect, the efficacy of Combined group was not limited in increasing oxygen saturation.

Another distinctive feature of hypoxic exposure is the activation of the immune-inflammatory responses, in which PDGF-AB is involved. We found that 6 h of hypoxia caused a significant neutrophil-dependent inflammatory response, which was consistent with previous studies [38, 39]. Particularly, our results showed that PDGF-AB, a downstream product of hypoxia-inducible factor (HIF) pathway, played a role during acute hypoxic exposure. PDGF family is composed of five dimeric isoforms (PDGF-AA, -AB, -BB, -CC, -DD) formed by four polypeptide chains (PDGF-A, -B, -C, -D) [40]. PDGF-A regulates the extracellular matrix and promotes collagen synthesis [41–43]. Its function is closely linked to the metalloproteinase family such as MMP and ADAM [44–46], as also shown in our study (Fig. 5d). PDGF-B functions mainly on vascular locations, recruits pericytes, and promotes vascular maturation [47–49].

**Fig. 8:**
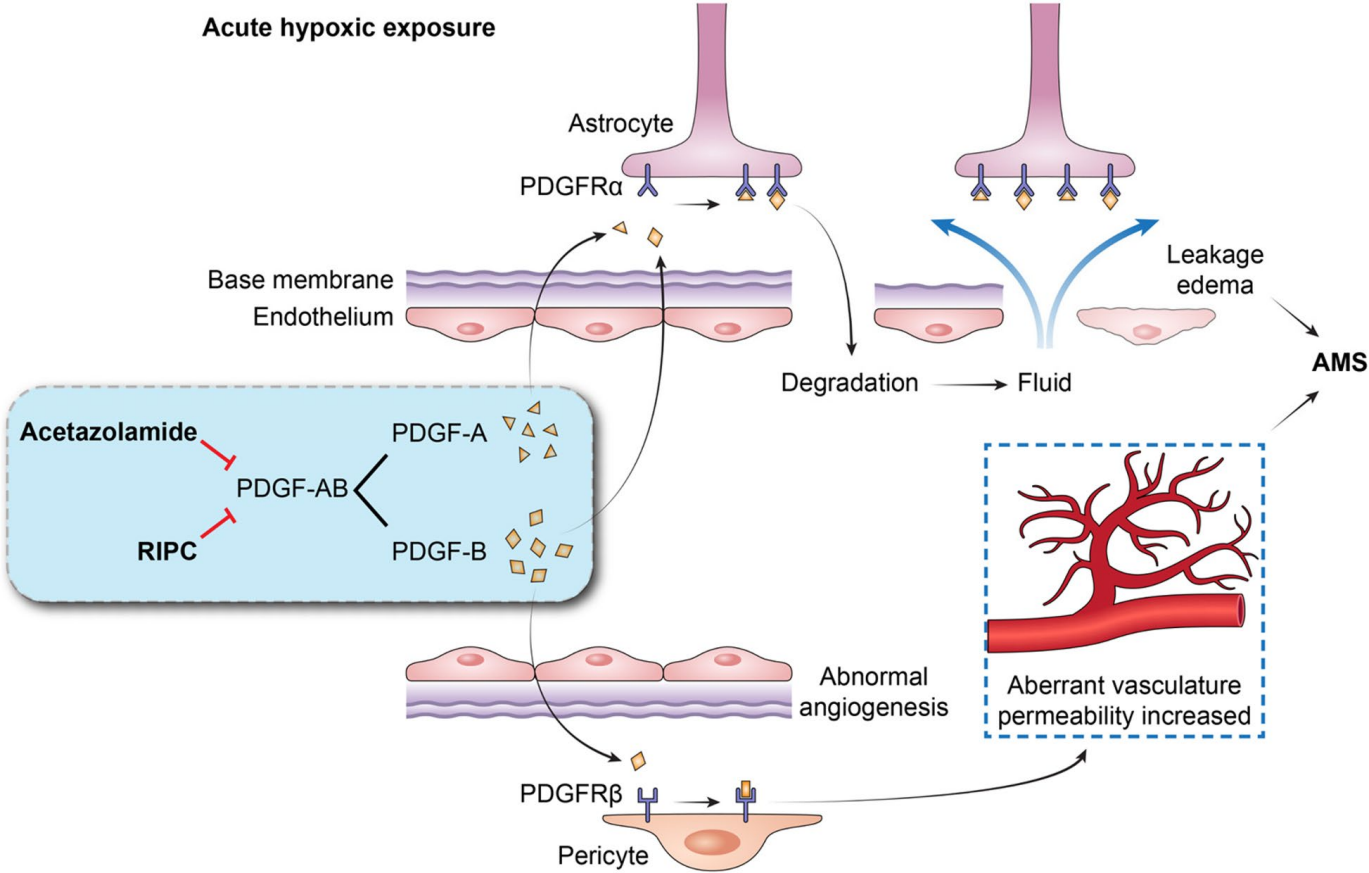
Hypothetical mechanism underlying the protection of vascular and barrier functions by the combination of acetazolamide and RIPC during hypoxic exposure. During hypoxia, PDGF-A interacts with PDGFRα distributed on perivascular regions. The activation of PDGFA/PDGFRα pathway results in an increase in MMP and ADAM expression. These enzymes break down the extracellular matrix collagen and degrade the tight junction proteins, leading to barrier dysfunction, leakage, edema, and ultimately, acute mountain sickness. PDGF-B also participates in PDGFRα pathway and interacts with PDGFRβ located on pericytes. Under hypoxic conditions, the activation of PDGF-B/PDGFRβ pathway leads to abnormal angiogenesis and aberrant vessel morphology. The permeability of the immature neogenetic vessel increases, which contributes to leakage and acute mountain sickness. Our findings revealed that before hypoxic exposure, acetazolamide, and RIPC synergistically reduce PDGF-AB levels, which probably “precondition” blood vessels prior to hypoxia, consequently minimizing the hypoxic damages

The enhanced effectiveness of the combination of acetazolamide and RIPC may result from a co-regulation of PDGF-AB, a factor that contributes to vascular and barrier dysfunction [50] and potentially causes AMS. Several studies have indicated elevated levels of PDGF-A and PDGF-B after hypoxic exposure [51, 52], which activates PDGFR in perivascular regions, initiating a cascade of subsequent events: MMP expression, breakdown of the extracellular matrix collagen, degradation of tight junction proteins, and disruption of vessel barriers, including the blood brain barrier (BBB) [45, 46, 50, 53]. We proposed that the downregulated of PDGF-AB by acetazolamide and RIPC intervention demonstrated in our study may block this process, “precondition” blood vessels prior to hypoxia, consequently minimizing the hypoxic damages and reducing the risk of AMS (Fig. 8). It should be noted that PDGF-AB levels did not elevate significantly after hypoxia in our study despite a trend of elevation (Fig. 7), which may be explained by limited exposure time. As the duration of hypoxic exposure increases (12–48 h), PDGF will rise significantly [51, 54].

We also demonstrated that *PDGFA* polymorphisms were associated with PDGF-AB concentration, risk of AMS, as well as the effectiveness to acetazolamide or RIPC. Previous studies have reported associations between genetic polymorphisms and AMS, with genes involved in the HIF signaling pathway genes being particularly highlighted., e.g., *EGLN1*, *EPAS1*, *PPARA*, and *VEGFA* [55–59]. Our study revealed that polymorphisms in *PDGFA*, also a downstream target gene of HIF, were associated with AMS susceptibility. The polymorphisms associated with AMS are located in non-coding regions and may influence protein expression. For example, a previous study focused on the treatment of tennis elbow with platelet-rich plasma (PRP) confirmed that *PDGFA* rs2070958 T carriers and rs1800814 A carriers showed lower PDGF-AB concentrations compared to CC or GG homozygotes [22]. Our study further demonstrated that rs2070958 T and rs1800814 A carriers responded to interventions with acetazolamide or RIPC and exhibited a significant reduction in PDGF-AB concentrations, indicating their potential role during acute hypoxic exposure.

Our study provides several novel insights. Firstly, we evaluated a novel and promising strategy for preventing AMS by combining acetazolamide and RIPC. Secondly, we explored the underlying mechanisms of their synergistic action, revealing the co-regulation of PDGF-AB by acetazolamide and RIPC. This discovery provides valuable insights into the physiological mechanisms and preventive strategies of AMS. Furthermore, we identified three genetic polymorphisms of *PDGFA* strongly associated with both AMS occurrence and intervention responsiveness, enhancing our understanding of AMS-related genes and providing a new framework for assessing risk in rapid-ascent populations.

Our study has several limitations that should be recognized. Firstly, due to inherent differences between the instrument and pharmacological intervention, we were unable to blind the subjects, which may introduce a potential placebo effect. Additionally, the 6-h duration of hypoxic exposure limited the long-term clinical and laboratory assessments. Finally, human trials are inevitably heterogeneous, emphasizing the need to confirm and explore detailed mechanisms using animal models and to conduct clinical trials in real altitude settings.

## Conclusions

The combination of acetazolamide and RIPC exerts a powerful anti-hypoxic effect and minimizes the risk of AMS. Acetazolamide improves oxygen saturation. RIPC further aids acetazolamide, which synergistically regulates PDGF-AB, potentially involved in the pathogenesis of AMS.

### Supplementary Information

**Additional file 1: Table S1.** Primer sequences for target SNPs. **Table S2.** Baseline demographic and clinical characteristics. **Figure S1.** AMS-Cerebral score and Chinese AMS score at 6 h of hypoxic exposure in different groups. **Figure S2.** Blood pressure and heart rate during hypoxic exposure. **Table S3.** Venous blood gas analysis of the five groups at baseline, pre-, and post-hypoxia. **Table S4.** Different characteristics between AMS (+) and AMS (−). **Table S5.** Differentially expressed proteins (DEPs) in Control group between baseline and post-hypoxia. **Table S6.** The overlapped proteins in DEPs group 1 (overlapped DEPs between baseline and pre-hypoxia in different intervention groups). **Table S7.** The overlapped proteins in DEPs group 2 (overlapped DEPs between baseline and post-hypoxia in different intervention groups). **Figure S3.** Analysis of PDGF-AB levels in AMS (+) and AMS (−) subjects. **Figure S4.** Analysis of PDGF-AB levels among different intervention groups based on AMS (−)/(+) subgroups. **Figure S5.** PDGF-BB levels in all subjects validated by ELISA. **Figure S6.** Analysis of PDGF-BB levels in AMS (+) and AMS (−) subjects. **Figure S7.** Analysis of PDGF-BB levels among different intervention groups based on AMS (−)/(+) subgroups. **Table S8.** Associations analyses between SNPs and AMS. **Table S9.** Decrease in PDGF-AB levels from baseline to pre-hypoxia in different SNP subgroups.

## Data Availability

The datasets used and/or analyzed during the current study are available from the corresponding author on reasonable request.

## Abbreviations

AMS: Acute mountain sickness
BBB: Blood brain barrier
BMI: Body mass index
CI: Confidence interval
DEP: Differentially expressed protein
EDTA: Ethylenediamine tetraacetic acid
ELISA: Enzyme-linked immunosorbent assay
HIF: Hypoxia-inducible factor
GO: Gene ontology
MAF: Minor allele frequency
NCBI: National Center for Biotechnology Information
OR: Odds ratio
PCR: Polymerase chain reaction
PDGF: Platelet-derived growth factor
PROBE: Prospective, randomized, open-label, blinded endpoint study
PRP: Platelet-rich plasma
RIPC: Remote ischemic preconditioning
SD: Standard deviation
SNP: Single-nucleotide polymorphisms
UTR: Untranslational region
