# Supplementary material for: Effects of acetazolamide combined with remote ischemic preconditioning on risk of acute mountain sickness: a randomized clinical trial
Source: BMC Med. 2024 Jan 2;22:4. doi: 10.1186/s12916-023-03209-7 (PMC10762951; doi:10.1186/s12916-023-03209-7)
Supplement: Supplementary file 1 — Additional file 1: Table S1. Primer sequences for target SNPs. Table S2. Baseline demographic and clinical characteristics. Figure S1. AMS-Cerebral score and Chinese AMS score at 6 h of hypoxic exposure in different groups. Figure S2. Blood pressure and heart rate during hypoxic exposure. Table S3. Venous blood gas analysis of the five groups at baseline, pre-, and post-hypoxia. Table S4. Different characteristics between AMS (+) and AMS (−). Table S5. Differentially expressed proteins (DEPs) in Control group between baseline and post-hypoxia. Table S6. The overlapped proteins in DEPs group 1 (overlapped DEPs between baseline and pre-hypoxia in different intervention groups). Table S7. The overlapped proteins in DEPs group 2 (overlapped DEPs between baseline and post-hypoxia in different intervention groups). Figure S3. Analysis of PDGF-AB levels in AMS (+) and AMS (−) subjects. Figure S4. Analysis of PDGF-AB levels among different intervention groups based on AMS (−)/(+) subgroups. Figure S5. PDGF-BB levels in all subjects validated by ELISA. Figure S6. Analysis of PDGF-BB levels in AMS (+) and AMS (−) subjects. Figure S7. Analysis of PDGF-BB levels among different intervention groups based on AMS (−)/(+) subgroups. Table S8. Associations analyses between SNPs and AMS. Table S9. Decrease in PDGF-AB levels from baseline to pre-hypoxia in different SNP subgroups. [file 12916_2023_3209_MOESM1_ESM.docx]

**Effects of Acetazolamide Combined with Remote Ischemic Preconditioning on Risk of Acute Mountain Sickness: A Randomized Clinical Trial**

**Additional File 1:**

[Table S1. Primer sequences for target SNPs. 2](#_Toc151991040)

[Table S2. Baseline demographic and clinical characteristics. 3](#_Toc151991041)

[Figure S1. AMS-Cerebral score and Chinese AMS score at six hours of hypoxic exposure in different groups. 4](#_Toc151991042)

[Figure S2. Blood pressure and heart rate during hypoxic exposure. 5](#_Toc151991043)

[Table S3. Venous blood gas analysis of the five groups at baseline, pre-, and post-hypoxia. 6](#_Toc151991044)

[Table S4. Different characteristics between AMS (+) and AMS (-). 6](#_Toc151991045)

[Table S5. Differentially expressed proteins (DEPs) in Control group between baseline and post-hypoxia. 8](#_Toc151991046)

[Table S6. The overlapped proteins in DEPs group 1 (overlapped DEPs between baseline and pre-hypoxia in different intervention groups). 9](#_Toc151991047)

[Table S7. The overlapped proteins in DEPs group 2 (overlapped DEPs between baseline and post-hypoxia in different intervention groups). 10](#_Toc151991048)

[Figure S3. Analysis of PDGF-AB levels in AMS (+) and AMS (-) subjects. 12](#_Toc151991049)

[Figure S4. Analysis of PDGF-AB levels among different intervention groups based on AMS (-)/(+) subgroups. 13](#_Toc151991050)

[Figure S5. PDGF-BB levels in all subjects validated by ELISA. 14](#_Toc151991052)

[Figure S6. Analysis of PDGF-BB levels in AMS (+) and AMS (-) subjects. 15](#_Toc151991053)

[Figure S7. Analysis of PDGF-BB levels among different intervention groups based on AMS (-)/(+) subgroups. 16](#_Toc151991054)

[Table S8. Associations analyses between SNPs and AMS. 17](#_Toc151991055)

[Table S9. Decrease in PDGF-AB levels from baseline to pre-hypoxia in different SNP subgroups. 19](#_Toc151991056)

# Table S1. Primer sequences for target SNPs.

| SNPs | Gene | Forward primer (5’ to 3’) | Reverse primer (5’ to 3’) |
| --- | --- | --- | --- |
| rs2070958 rs1800814  rs1088814 | *PDGFA* | CCGAGAAACTTCTGAGTCCC | TACACTCCCCACCACAAGGTA |
| rs9690350 | *PDGFA* | CCGGACTCAGAGCTCACAA | CCTAAGCCTCCTGAATGCTAC |
| rs62433334 | *PDGFA* | TGGCACTTGACACTGCTCGT | ATTTTGCACCCTTCAGACCACT |
| rs1800817 | *PDGFB* | CCAGATTCGCCCGCCGGTTG | CTCTGCTGCTACCTGCGTCT |
| rs1800818 | *PDGFB* | AAAAGGAACACGGCAGTCGAT | TCCACCCACCTCTCGCACT |
| rs2285099  rs2285094 | *PDGFB* | AAAAGAAAGACCTCGTCAGC | ACCATTTCTCAGAGTGGCAAG |

# Table S2. Baseline demographic and clinical characteristics.

| Characteristics | Groups | | | | | *P* value |
| --- | --- | --- | --- | --- | --- | --- |
|  | Control group | Ripc group | Rapid-Ripc group | Acetazolamide group | Combined group |  |
| Age (years) | 30.04 ± 7.11 | 30.62 ± 7.21 | 30.96 ± 7.27 | 31.14 ± 6.64 | 29.16 ± 7.60 | 0.644 |
| Male, No. (%) | 21 (42.0) | 20 (40.0) | 22 (44.0) | 16 (32.0) | 19 (38.0) | 0.776 |
| Weight (kg) | 65.69 ± 15.71 | 65.82 ± 13.76 | 65.02 ± 11.72 | 62.33 ± 11.33 | 64.35 ± 14.29 | 0.703 |
| Body mass index (kg/m^2^) | 22.99 ± 3.91 | 21.76 ± 7.55 | 23.04 ± 2.30 | 22.55 ± 3.10 | 22.56 ± 3.95 | 0.653 |
| Physical activity, No. (%) | 8 (16.0) | 9 (18.0) | 12 (24.0) | 10 (20.0) | 11 (22.0) | 0.870 |
| Previously reached the plateau, No. (%) | 11 (22.0) | 9 (18.0) | 15 (30.0) | 9 (18.0) | 12 (24.0) | 0.583 |
| Previous discomfort at plateau, No. (%) | 4 (8.0) | 5 (10.0) | 9 (18.0) | 3 (6.0) | 4 (8.0) | 0.299 |
| Systolic blood pressure (mm Hg) | 122.40 ± 15.38 | 122.80 ± 15.91 | 122.30 ± 14.37 | 118.30 ± 12.03 | 120.10 ± 12.58 | 0.452 |
| Diastolic blood pressure (mm Hg) | 79.34 ± 12.77 | 74.44 ± 12.57 | 74.76 ± 11.57 | 73.38 ± 11.12 | 74.68 ± 12.00 | 0.135 |
| Heart rate (beats per minute) | 83.60 ± 11.97 | 80.34 ± 11.49 | 78.42 ± 13.08 | 78.22 ± 10.58 | 79.34 ± 10.28 | 0.144 |
| SpO_2_ (%) | 98.77 ± 0.89 | 98.40 ± 1.11 | 98.40 ± 1.26 | 98.56 ± 1.07 | 98.46 ± 1.05 | 0.429 |
| HAMA | 3.18 ± 4.01 | 2.98 ± 3.31 | 2.67 ± 3.50 | 1.94 ± 2.07 | 3.04 ± 4.01 | 0.399 |
| HAMD | 1.98 ± 2.37 | 2.18 ± 2.58 | 1.73 ± 2.39 | 1.35 ± 1.72 | 1.82 ± 2.51 | 0.479 |

HAMA, Hamilton Anxiety Scale; HAMD, Hamilton Depression Scale; Regular physical activity was defined as exercising more than three times a week for more than 30 minutes each time or engaging in physical labor. Data are no. (%) or mean with SD.


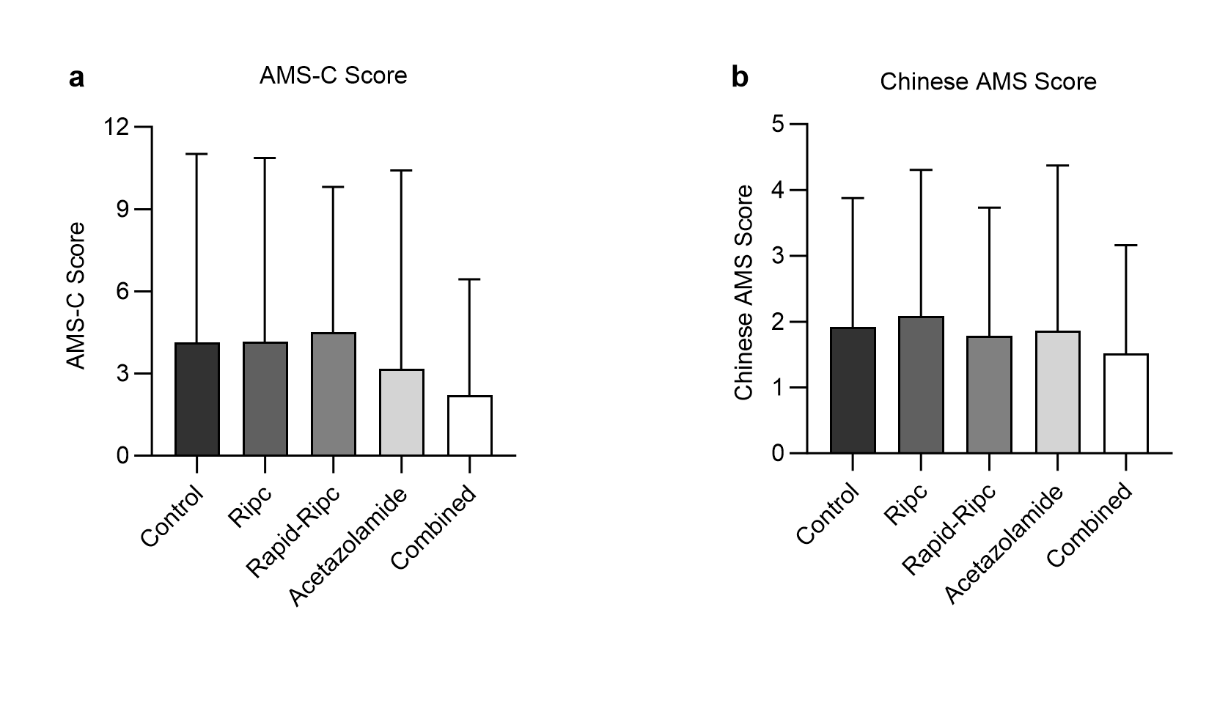


Figure S1. AMS-Cerebral score and Chinese AMS score at six hours of hypoxic exposure in different groups. (a) AMS-Cerebral score evaluated by Environmental Symptoms Questionnaire-III (ESQ-III). The AMS-Cerebral score in Control, Ripc, Rapid-Ripc, Acetazolamide, and Combined group were 4.14 ± 6.87, 4.16 ± 6.17, 4.51 ± 5.30, 3.16 ± 7.26, 2.20 ± 4.24, respectively. No significant differences were found between groups (*P* = 0.083). (b) Chinese AMS score. Chinese AMS score of the five groups were 1.92 ± 1.96, 2.08 ± 2.23, 1.79 ± 1.94, 1.86 ± 2.52, 1.52 ± 1.64, respectively. No significant differences were found between groups (*P* = 0.800). Data are mean with SD.


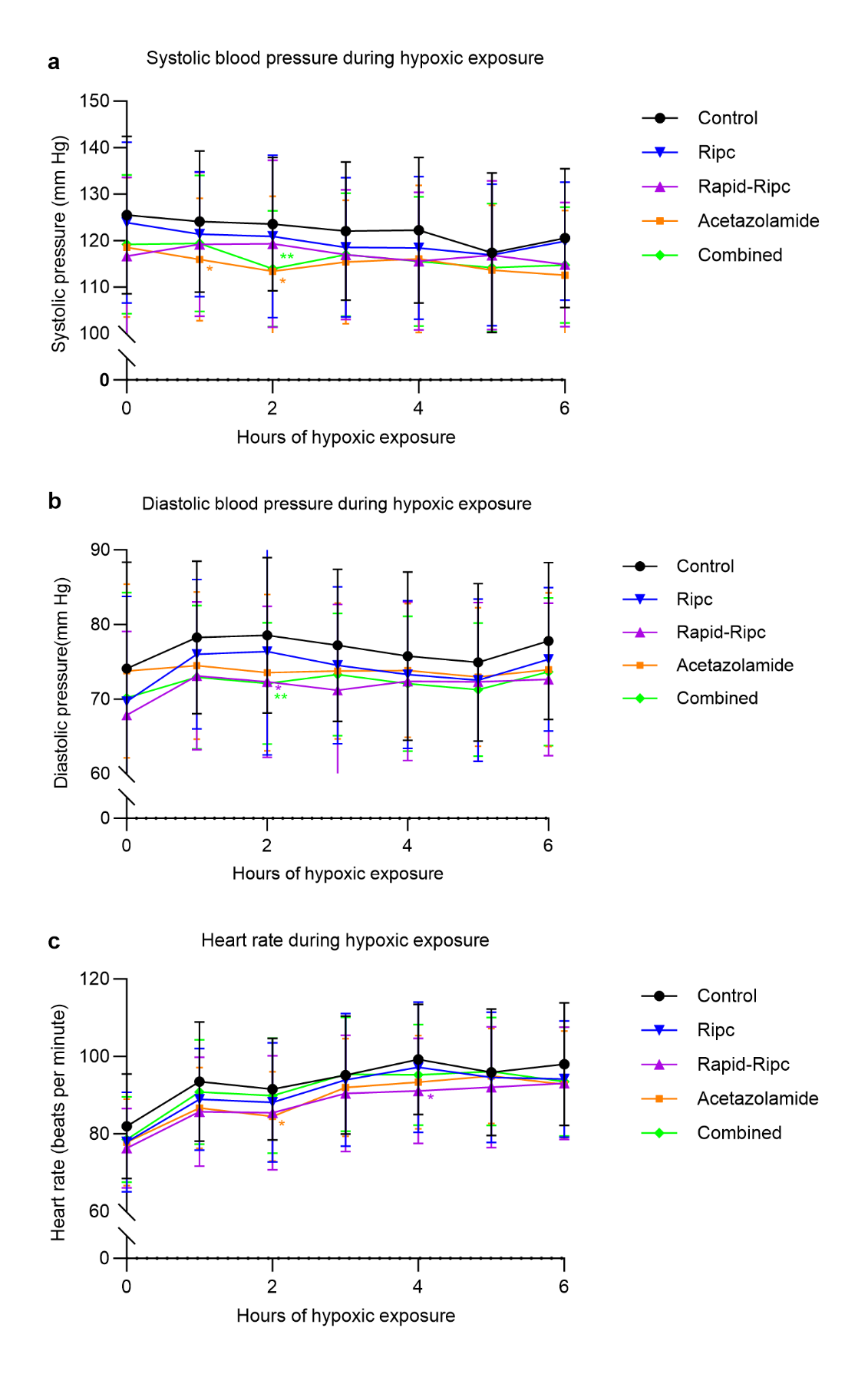


Figure S2. Blood pressure and heart rate during hypoxic exposure. (a) Systolic blood pressure and (b) diastolic blood pressure fluctuated little during the hypoxic exposure. (c) Heart rate increased mildly after one hour of hypoxic exposure and then fluctuated a little. Data are mean with SD. ^*^*P* < 0.05, ^**^*P* < 0.01 compared with Control group.

# Table S3. Venous blood gas analysis of the five groups at baseline, pre-, and post-hypoxia.

|  | Time points of hypoxic exposure | | | *P* value | | |
| --- | --- | --- | --- | --- | --- | --- |
|  | baseline | pre-hypoxia | post-hypoxia | baseline vs pre-hypoxia | pre- vs post-hypoxia | baseline vs post-hypoxia |
| pH |  |  |  |  |  |  |
| Control | 7.34 ± 0.04 | 7.36 ± 0.03 | 7.38 ± 0.03 | 0.918 | 0.750 | 0.825 |
| Ripc | 7.36 ± 0.05 | 7.35 ± 0.04 | 7.39 ± 0.04 | 0.631 | <0.001 | 0.015 |
| Rapid-Ripc | 7.37 ± 0.04 | 7.36 ± 0.04 | 7.38 ± 0.03 | 0.994 | 0.180 | 0.312 |
| Acetazolamide | 7.38 ± 0.04 | 7.30 ± 0.04^**^ | 7.31 ± 0.03^****^ | <0.001 | 0.236 | <0.001 |
| Combined | 7.38 ± 0.05 | 7.31 ± 0.07^*^ | 7.33 ± 0.04^****^ | <0.001 | 0.261 | <0.001 |
| P50 (mm Hg) |  |  |  |  |  |  |
| Control | 27.83 ± 1.20 | 27.67 ± 0.97 | 27.56 ± 1.13 | 0.999 | 0.999 | 0.748 |
| Ripc | 28.09 ± 1.38 | 28.47 ± 1.21 | 27.38 ± 1.26 | 0.605 | <0.001 | 0.009 |
| Rapid-Ripc | 27.95 ± 1.19 | 28.37 ± 1.30 | 27.49 ± 1.08 | 0.486 | 0.001 | 0.162 |
| Acetazolamide | 27.69 ± 1.30 | 29.70 ± 1.36^**^ | 29.26 ± 1.21^****^ | <0.001 | 0.239 | <0.001 |
| Combined | 27.73 ± 1.30 | 29.51 ± 2.32^**^ | 28.70 ± 1.43^**^ | <0.001 | 0.003 | <0.001 |
| Actual bicarbonate (mmol/L) |  |  |  |  |  |  |
| Control | 27.14 ± 5.92 | 26.98 ± 5.93 | 26.30 ± 2.57 | 0.292 | 0.494 | 0.762 |
| Ripc | 26.43 ± 3.73 | 26.36 ± 3.55 | 26.11 ± 3.60 | 0.999 | 0.999 | 0.995 |
| Rapid-Ripc | 26.30 ± 3.15 | 29.56 ± 2.92 | 25.83 ± 3.62 | 0.023 | 0.003 | 0.985 |
| Acetazolamide | 25.47 ± 3.09 | 19.93 ± 3.16^****^ | 20.75 ± 2.69^****^ | 0.001 | 0.991 | <0.001 |
| Combined | 26.13 ± 4.78 | 23.38 ± 4.38^*^ | 20.21 ± 3.39^****^ | 0.076 | 0.015 | <0.001 |
| Standard bicarbonate (mmol/L) |  |  |  |  |  |  |
| Control | 23.58 ± 2.46 | 21.93 ± 0.97 | 23.37 ± 1.38 | 0.556 | 0.628 | 0.992 |
| Ripc | 23.44 ± 2.56 | 22.95 ± 2.11 | 23.31 ± 1.87 | 0.952 | 0.975 | 0.999 |
| Rapid-Ripc | 23.40 ± 1.83 | 25.12 ± 1.71 | 23.30 ± 1.74 | 0.047 | 0.011 | 0.999 |
| Acetazolamide | 23.21 ± 2.13 | 18.12 ± 2.74^**^ | 18.56 ± 1.66^****^ | <0.001 | 0.946 | <0.001 |
| Combined | 23.33 ± 3.35 | 20.84 ± 2.16 | 18.72 ± 1.92^****^ | 0.001 | 0.001 | <0.001 |
| Base excess (mmol/L) |  |  |  |  |  |  |
| Control | 0.75 ± 3.37 | -1.41 ± 1.16 | 0.87 ± 2.23 | 0.158 | 0.012 | 0.999 |
| Ripc | 0.50 ± 3.44 | 0.17 ± 2.90 | 0.67 ± 2.77 | 0.978 | 0.673 | 0.998 |
| Rapid-Ripc | 0.53 ± 2.57 | -0.44 ± 2.21 | 0.81 ± 2.45 | 0.390 | 0.141 | 0.994 |
| Acetazolamide | -0.01 ± 2.89 | -6.07 ± 3.50^***^ | -5.21 ± 2.23^****^ | <0.001 | 0.160 | <0.001 |
| Combined | 0.49 ± 4.31 | -5.85 ± 2.78^***^ | -5.22 ± 2.73^****^ | <0.001 | 0.917 | <0.001 |
| Lactate (mmol/L) |  |  |  |  |  |  |
| Control | 1.39 ± 0.36 | 1.55 ± 0.46 | 1.87 ± 0.40 | 0.731 | 0.242 | <0.001 |
| Ripc | 1.48 ± 0.40 | 1.56 ± 0.35 | 1.93 ± 0.63 | 0.770 | <0.001 | <0.001 |
| Rapid-Ripc | 1.44 ± 0.34 | 1.74 ± 0.47 | 1.74 ± 0.30 | <0.001 | 0.999 | <0.001 |
| Acetazolamide | 1.35 ± 0.43 | 1.51 ± 0.41 | 1.76 ± 0.41 | 0.073 | 0.007 | <0.001 |
| Combined | 1.40 ± 0.45 | 1.68 ± 0.50 | 1.86 ± 0.53 | <0.001 | 0.080 | <0.001 |

# Data are mean ± SD. ^*^*P* < 0.05 with Control group, ^**^*P* < 0.01, ^***^*P* < 0.001, ^****^*P* < 0.0001.Table S4. Different characteristics between AMS (+) and AMS (-).

|  | All | AMS (+) | AMS (-) | *P* value |
| --- | --- | --- | --- | --- |
| Total, No. (%) | 250 | 48 (19.2) | 202 (80.8) | - |
| AMS score at six hours of hypoxic exposure | 1.48 ± 1.71 | 4.17 ± 1.79 | 0.84 ± 0.86 | <0.001 |
| Participant characteristics |  |  |  |  |
| Age (years) | 30.38 ± 7.15 | 29.75 ± 6.61 | 30.53 ± 7.28 | 0.495 |
| Male, No. (%) | 98 (39.2) | 14 (29.2) | 84 (41.6) | 0.113 |
| Body mass index (kg/m^2^) | 22.58 ± 4.59 | 23.44 ± 3.91 | 22.37 ± 4.73 | 0.150 |
| Physical activity, No. (%) | 50 (20.0) | 9 (18.8) | 41 (20.3) | 0.845 |
| Previous discomfort at plateau, No. (%) | 25 (10.0) | 8 (16.7) | 17 (8.4) | 0.107 |
| HAMA scale | 2.87 ± 3.80 | 3.67 ± 5.18 | 2.68 ± 3.40 | 0.220 |
| HAMD scale | 1.87 ± 2.52 | 2.50 ± 3.21 | 1.73 ± 2.32 | 0.129 |
| Vital signs at six hours of hypoxic exposure |  |  |  |  |
| Systolic blood pressure (mm Hg) | 116.49 ± 13.76 | 115.26 ± 13.83 | 116.78 ± 13.77 | 0.505 |
| Diastolic blood pressure (mm Hg) | 74.67 ± 14.21 | 73.67 ± 13.58 | 74.90 ± 10.14 | 0.464 |
| Heart rate (beats per minute) | 94.32 ± 14.68 | 94.04 ± 14.73 | 94.38 ± 14.70 | 0.888 |
| SpO_2_ (%) | 87.30 ± 5.85 | 84.89 ± 6.03 | 87.86 ± 5.68 | 0.002 |
| Blood count at six hours of hypoxic exposure |  |  |  |  |
| White blood cell count (×10^9^/L) | 7.04 ± 1.71 | 7.26 ± 2.00 | 6.98 ± 1.64 | 0.684 |
| Percentage of neutrophils (%) | 62.38 ± 8.98 | 65.58 ± 11.12 | 61.64 ± 8.26 | 0.027 |
| Percentage of lymphocytes (%) | 29.92 ± 7.79 | 27.61 ± 9.46 | 30.45 ± 7.27 | 0.061 |
| Percentage of monocytes (%) | 5.44 ± 1.60 | 4.89 ± 1.79 | 5.57 ± 1.53 | 0.005 |
| Neutrophil count (×10^9^/L) | 4.45 ± 1.47 | 4.87 ± 1.93 | 4.35 ± 1.33 | 0.191 |
| Lymphocyte count (×10^9^/L) | 2.06 ± 0.63 | 1.92 ± 0.66 | 2.09 ± 0.61 | 0.098 |
| Neutrophil-lymphocyte ratio | 2.42 ± 1.63 | 3.03 ± 2.27 | 2.29 ± 1.42 | 0.036 |
| Monocyte count (×10^9^/L) | 0.37 ± 0.11 | 0.34 ± 0.10 | 0.38 ± 0.12 | 0.022 |
| Red blood cell count (×10^12^/L) | 4.85 ± 0.47 | 4.75 ± 0.44 | 4.87 ± 0.48 | 0.113 |
| Hemoglobin (g/L) | 147.18 ± 18.25 | 142.80 ± 18.37 | 148.20 ± 18.12 | 0.071 |
| Platelet count (×10^9^/L) | 265.28 ± 67.62 | 278.61 ± 92.79 | 262.20 ± 60.22 | 0.138 |
| Venous blood gas at pre-hypoxia |  |  |  |  |
| pH | 7.33 ± 0.06 | 7.35 ± 0.06 | 7.33 ± 0.06 | 0.123 |
| P50 (mm Hg) | 28.96 ± 1.70 | 28.71 ± 1.39 | 29.01 ± 1.76 | 0.324 |
| Actual bicarbonate (mmol/L) | 24.74 ± 6.74 | 26.99 ± 8.54 | 24.26 ± 6.24 | 0.025 |
| Standard bicarbonate (mmol/L) | 21.79 ± 4.73 | 23.41 ± 6.01 | 21.44 ± 4.37 | 0.022 |
| Base excess (mmol/L) | -2.88 ± 4.03 | -2.03 ± 4.03 | -3.05 ± 4.04 | 0.192 |
| Lactate (mmol/L) | 1.62 ± 0.44 | 1.64 ± 0.41 | 1.61 ± 0.45 | 0.666 |

Data are no. (%) or mean with SD.

# Table S5. Differentially expressed proteins (DEPs) in Control group between baseline and post-hypoxia.

| Protein | Full name | *P* value | Fold change | Regulation |
| --- | --- | --- | --- | --- |
| CCL23 | C-C motif chemokine ligand 23 | 0.004 | 278.21 | up |
| ADAM12 | Disintegrin and metalloproteinase domain-containing protein 12 | <0.001 | 166.52 | up |
| DR3 | Tumor necrosis factor receptor superfamily member 25 | 0.001 | 152.70 | up |
| ICOS | Inducible T-cell costimulator | 0.005 | 93.02 | up |
| ULBP-1 | UL16-binding protein 1 | 0.013 | 55.85 | up |
| ADAM8 | Disintegrin and metalloproteinase domain-containing protein 8 | 0.018 | 47.99 | up |
| BMPR-1B | Bone morphogenetic protein receptor type-1B | 0.016 | 42.25 | up |
| Siglec-10 | Sialic acid-binding Ig-like lectin 10 | 0.013 | 42.08 | up |
| S100A8 | Protein S100-A8 | 0.021 | 40.33 | up |
| CD48 | CD48 antigen | 0.019 | 39.56 | up |
| IL-17E | Interleukin-25 | 0.048 | 37.20 | up |
| JAM-B | Junctional adhesion molecule B | 0.020 | 36.32 | up |
| Tie-1 | Tyrosine kinase with immunoglobulin and epidermal growth factor homology domains 1 | 0.034 | 34.05 | up |
| OSM | Oncostatin-M | 0.049 | 21.69 | up |
| TACE | Disintegrin and metalloproteinase domain-containing protein 17 | 0.018 | 18.46 | up |
| ErbB4 | Receptor tyrosine-protein kinase erbB-4 | 0.041 | 15.20 | up |
| IL-32α | Interleukin-32 | 0.028 | 12.54 | up |
| SLAM | Signaling lymphocytic activation molecule | 0.048 | 7.14 | up |
| Testican 2 | Testican-2 | 0.001 | 2.98 | up |
| Cystatin A | Cystatin-A | 0.015 | 2.20 | up |
| MMP-8 | Matrix metalloproteinase-8 | 0.003 | 2.10 | up |
| JAM-A | Junctional adhesion molecule A | 0.010 | 2.08 | up |
| Lymphotactin | Lymphotactin | 0.002 | 0.50 | down |
| DAN | Poly(A)-specific ribonuclease PARN | 0.001 | 0.49 | down |
| GRO | Growth-regulated alpha protein | 0.008 | 0.49 | down |
| IL-1β | Interleukin-1 beta | 0.030 | 0.47 | down |
| IGFBP-1 | Insulin-like growth factor-binding protein 1 | 0.004 | 0.46 | down |
| PDGF-BB | Platelet-derived growth factor-BB | 0.022 | 0.45 | down |
| IL-4 | Interleukin-34 | 0.002 | 0.39 | down |
| PDGF-AB | Platelet-derived growth factor-AB | 0.003 | 0.34 | down |

# Table S6. The overlapped proteins in DEPs group 1 (overlapped DEPs between baseline and pre-hypoxia in different intervention groups).

|  |  | AveExp. pre | AveExp. base | logFC | P.Val | FC | Reg. | S1-pre | S2-pre | S3-pre | S4-pre | S5-pre | S6-pre | S1-base | S2-base | S3-base | S4-base | S5-base | S6-base | Ave.S | Ave.all | Reasons for not validating |
| --- | --- | --- | --- | --- | --- | --- | --- | --- | --- | --- | --- | --- | --- | --- | --- | --- | --- | --- | --- | --- | --- | --- |
| 1 | **IL32α** | | | | | | | | | | | | | | | | | | | | | |
|  | Control | - | | | | | | | | | | | | | | | | | | | | low intensity^1^ |
|  | Acetazolamide | 7.65 | 1.46 | 6.19 | 0.01 | 72.88 | up | 386.40 | 214.38 | 1085.19 | 0.00 | 759.66 | 947.03 | 0.00 | 0.00 | 0.00 | 0.00 | 435.08 | 0.00 | 318.98 | 384.21 |  |
|  | Rapid-Ripc | 8.42 | 3.00 | 5.42 | 0.02 | 42.82 | up | 111.43 | 444.00 | 292.71 | 319.58 | 558.64 | 620.57 | 327.34 | 0.00 | 0.00 | 0.00 | 808.98 | 0.00 | 290.27 |  |  |
|  | Combined | 7.62 | 3.38 | 4.24 | 0.04 | 18.86 | up | 339.57 | 986.90 | 0.00 | 110.97 | 1252.46 | 1205.73 | 0.00 | 634.85 | 0.00 | 0.00 | 1990.27 | 0.00 | 543.40 |  |  |
| 2 | **ADAM8** | | | | | | | | | | | | | | | | | | | | | |
|  | Control | - | | | | | | | | | | | | | | | | | | | | low intensity |
|  | Acetazolamide | 9.82 | 5.01 | 4.81 | 0.02 | 28.02 | up | 1134.32 | 2235.36 | 297.11 | 503.61 | 991.89 | 1430.92 | 0.00 | 1620.98 | 0.00 | 0.00 | 490.40 | 1407.73 | 842.69 | 831.22 |  |
|  | Rapid-Ripc | 9.74 | 3.30 | 6.43 | 0.01 | 86.47 | up | 1242.42 | 1183.07 | 747.69 | 464.99 | 1627.71 | 461.89 | 1101.89 | 0.00 | 0.00 | 0.00 | 839.07 | 0.00 | 639.06 |  |  |
|  | Combined | 10.05 | 8.22 | 1.83 | 0.21 | 3.55 | up | 438.20 | 1319.18 | 1481.85 | 1115.19 | 2184.66 | 683.03 | 474.06 | 973.70 | 1252.14 | 1208.06 | 1012.78 | 0.00 | 1011.91 |  |  |
| 3 | **ADAM12** | | | | | | | | | | | | | | | | | | | | | |
|  | Control | - | | | | | | | | | | | | | | | | | | | | low intensity |
|  | Acetazolamide | 7.22 | 3.87 | 3.35 | 0.05 | 10.20 | up | 832.25 | 314.17 | 0.00 | 96.18 | 969.93 | 451.42 | 0.00 | 275.03 | 0.00 | 0.00 | 379.40 | 93.53 | 284.33 | 438.11 |  |
|  | Rapid-Ripc | 9.05 | 2.54 | 6.51 | 0.01 | 90.93 | up | 123.33 | 113.07 | 1647.26 | 1165.16 | 1320.94 | 607.39 | 93.50 | 0.00 | 0.00 | 0.00 | 409.49 | 0.00 | 456.68 |  |  |
|  | Combined | 9.35 | 6.20 | 3.15 | 0.10 | 8.86 | up | 834.24 | 389.95 | 981.42 | 583.80 | 821.52 | 491.52 | 1188.42 | 756.38 | 426.76 | 0.00 | 405.90 | 0.00 | 573.33 |  |  |
| 4 | **JAM-B** | | | | | | | | | | | | | | | | | | | | | |
|  | Control | - | | | | | | | | | | | | | | | | | | | | low intensity |
|  | Acetazolamide | 9.01 | 5.62 | 3.39 | 0.04 | 10.46 | up | 364.28 | 1330.43 | 332.12 | 283.11 | 748.50 | 538.53 | 0.00 | 693.71 | 186.12 | 0.00 | 406.01 | 267.21 | 429.17 | 519.08 |  |
|  | Rapid-Ripc | 8.99 | 2.96 | 6.03 | 0.01 | 65.31 | up | 390.76 | 594.05 | 485.27 | 317.07 | 1210.28 | 400.35 | 375.62 | 0.00 | 0.00 | 0.00 | 598.60 | 0.00 | 364.33 |  |  |
|  | Combined | 9.67 | 7.58 | 2.09 | 0.15 | 4.25 | up | 370.33 | 710.55 | 437.74 | 672.13 | 1409.52 | 2608.97 | 287.93 | 730.49 | 420.34 | 0.00 | 938.66 | 578.03 | 763.73 |  |  |
| 5 | **Siglec-10** | | | | | | | | | | | | | | | | | | | | | |
|  | Control | - | | | | | | | | | | | | | | | | | | | | low intensity |
|  | Acetazolamide | 9.09 | 5.85 | 3.23 | 0.05 | 9.41 | up | 407.93 | 776.43 | 362.19 | 194.49 | 1167.02 | 982.20 | 0.00 | 529.45 | 282.01 | 0.00 | 672.28 | 368.93 | 478.58 | 506.13 |  |
|  | Rapid-Ripc | 9.29 | 5.53 | 3.76 | 0.04 | 13.56 | up | 358.20 | 250.15 | 1012.88 | 810.38 | 828.53 | 988.43 | 321.50 | 0.00 | 509.80 | 0.00 | 277.89 | 212.59 | 464.19 |  |  |
|  | Combined | 9.46 | 7.07 | 2.39 | 0.10 | 5.23 | up | 405.06 | 453.90 | 1240.42 | 653.98 | 1058.75 | 759.51 | 211.35 | 671.32 | 824.90 | 0.00 | 535.51 | 92.58 | 575.61 |  |  |
| 6 | **ULBP-1** | | | | | | | | | | | | | | | | | | | | | |
|  | Control | - | | | | | | | | | | | | | | | | | | | | low intensity |
|  | Acetazolamide | 10.05 | 6.43 | 3.61 | 0.04 | 12.25 | up | 974.58 | 734.54 | 1145.56 | 204.31 | 2635.40 | 3176.81 | 0.00 | 406.42 | 1016.78 | 0.00 | 1581.26 | 637.00 | 1042.72 | 926.13 |  |
|  | Rapid-Ripc | 10.00 | 4.95 | 5.05 | 0.03 | 33.22 | up | 1251.39 | 474.68 | 777.87 | 1221.79 | 2115.21 | 970.16 | 1992.97 | 0.00 | 0.00 | 0.00 | 787.22 | 551.39 | 845.22 |  |  |
|  | Combined | 10.10 | 9.01 | 1.08 | 0.03 | 2.12 | up | 782.57 | 655.92 | 1282.08 | 777.59 | 1446.50 | 2305.88 | 834.85 | 724.08 | 546.64 | 202.99 | 756.96 | 369.15 | 890.43 |  |  |

AveExp, average expression, calculated as average of log_2_(protein intensity+1); AveExp. pre: average expression for pre-hypoxia; AveExp.base, average expression for baseline; P Val, P value; FC, foldchange; Reg, regulation; S, subject; Ave.S, average intensity within each intervention group; Ave all, average intensity in all intervention groups. 1 Intensity greater than 1500 is considered suitable for further validation.

# Table S7. The overlapped proteins in DEPs group 2 (overlapped DEPs between baseline and post-hypoxia in different intervention groups).

|  |  | AveExp. post | AveExp. base | logFC | P.Val | FC | Reg. | S1-post | S2-post | S3-post | S4-post | S5-post | S6-post | S1-base | S2-base | S3-base | S4-base | S5-base | S6-base | Ave.S | Ave.all | Reasons for not validating |
| --- | --- | --- | --- | --- | --- | --- | --- | --- | --- | --- | --- | --- | --- | --- | --- | --- | --- | --- | --- | --- | --- | --- |
| 1 | **IGFBP-1** | | | | | | | | | | | | | | | | | | | | | |
|  | Control | 12.14 | 13.24 | -1.09 | 0.00 | 0.47 | down | 3466.01 | 2482.21 | 5780.43 | 5573.81 | 8403.29 | 3691.07 | 4580.50 | 5472.32 | 14153.09 | 11357.83 | 39202.02 | 5125.98 | 9107.38 | 7216.75 | circadien variation^1^ |
|  | Acetazolamide | 11.92 | 12.95 | -1.02 | 0.01 | 0.49 | down | 2065.88 | 2610.02 | 8279.42 | 3539.84 | 3743.82 | 5795.88 | 4393.64 | 7928.95 | 6376.72 | 6562.89 | 9658.97 | 17210.64 | 6513.89 |  |  |
|  | Rapid-Ripc | 12.01 | 13.12 | -1.11 | 0.00 | 0.46 | down | 2313.48 | 3077.47 | 4552.07 | 7241.24 | 5043.83 | 4163.62 | 8565.74 | 6831.69 | 12709.47 | 20008.27 | 4959.39 | 6633.11 | 7174.95 |  |  |
|  | Combined | 11.73 | 12.75 | -1.02 | 0.01 | 0.49 | down | 2425.51 | 1880.48 | 2549.06 | 3731.15 | 6021.97 | 5782.28 | 5776.96 | 2554.21 | 11751.07 | 3403.98 | 11496.33 | 15476.35 | 6070.78 |  |  |
| 2 | **DR3** | | | | | | | | | | | | | | | | | | | | | |
|  | Control | 8.52 | 1.26 | 7.25 | 0.00 | 152.70 | up | 416.03 | 305.92 | 516.03 | 188.35 | 283.42 | 676.46 | 0.00 | 189.48 | 0.00 | 0.00 | 0.00 | 0.00 | 214.64 | 202.07 | low intensity^2^ |
|  | Acetazolamide | 6.83 | 2.66 | 4.17 | 0.02 | 18.04 | up | 0.00 | 299.35 | 106.22 | 872.78 | 288.90 | 264.54 | 0.00 | 291.06 | 0.00 | 214.24 | 0.00 | 0.00 | 194.76 |  |  |
|  | Rapid-Ripc | 7.15 | 2.35 | 4.79 | 0.02 | 27.76 | up | 397.93 | 200.76 | 388.55 | 480.24 | 532.99 | 0.00 | 85.37 | 0.00 | 0.00 | 0.00 | 202.94 | 0.00 | 190.73 |  |  |
|  | Combined | 8.08 | 3.91 | 4.17 | 0.02 | 18.02 | up | 453.35 | 352.60 | 277.77 | 157.75 | 358.77 | 153.11 | 0.00 | 371.04 | 121.43 | 0.00 | 251.88 | 0.00 | 208.14 |  |  |
| 3 | **IL32α** | | | | | | | | | | | | | | | | | | | | | |
|  | Control | 9.44 | 5.80 | 3.65 | 0.03 | 12.54 | up | 241.48 | 341.44 | 4281.86 | 839.92 | 1348.01 | 283.03 | 0.00 | 503.14 | 2061.77 | 89.42 | 312.78 | 0.00 | 858.57 | 524.12 | low intensity |
|  | Acetazolamide | 7.99 | 1.46 | 6.53 | 0.01 | 92.44 | up | 410.86 | 348.94 | 1117.58 | 0.00 | 1162.05 | 1450.08 | 0.00 | 0.00 | 0.00 | 0.00 | 435.08 | 0.00 | 410.38 |  |  |
|  | Rapid-Ripc | 6.14 | 3.00 | 3.13 | 0.10 | 8.76 | up | 528.48 | 0.00 | 505.54 | 503.38 | 890.07 | 0.00 | 327.34 | 0.00 | 0.00 | 0.00 | 808.98 | 0.00 | 296.98 |  |  |
|  | Combined | 8.95 | 3.38 | 5.57 | 0.01 | 47.43 | up | 309.26 | 438.48 | 389.11 | 240.02 | 1707.45 | 657.22 | 0.00 | 634.85 | 0.00 | 0.00 | 1990.27 | 0.00 | 530.56 |  |  |
| 4 | **ErbB4** | | | | | | | | | | | | | | | | | | | | | |
|  | Control | 9.61 | 5.69 | 3.93 | 0.04 | 15.20 | up | 1041.99 | 911.98 | 809.87 | 557.47 | 857.04 | 618.82 | 272.36 | 569.36 | 0.00 | 289.47 | 410.27 |  | 576.24 | 556.43 | low intensity |
|  | Acetazolamide | 9.37 | 5.88 | 3.50 | 0.05 | 11.28 | up | 356.08 | 1392.03 | 789.84 | 556.02 | 564.00 | 690.04 | 0.00 | 963.45 | 352.20 | 0.00 | 317.62 | 382.08 | 530.28 |  |  |
|  | Rapid-Ripc | 9.43 | 3.03 | 6.39 | 0.01 | 84.03 | up | 821.08 | 837.05 | 678.17 | 754.78 | 1193.12 | 251.06 | 405.93 | 0.00 | 0.00 | 0.00 | 741.87 | 0.00 | 473.59 |  |  |
|  | Combined | 9.73 | 7.35 | 2.39 | 0.11 | 5.23 | up | 778.01 | 808.44 | 987.64 | 907.61 | 873.82 | 765.72 | 207.34 | 971.61 | 468.94 | 0.00 | 700.55 | 277.44 | 645.59 |  |  |
| 5 | **ADAM8** | | | | | | | | | | | | | | | | | | | | | |
|  | Control | 10.02 | 4.44 | 5.58 | 0.02 | 47.99 | up | 1445.36 | 1351.49 | 1235.64 | 681.13 | 1044.17 | 739.51 | 0.00 | 1016.49 | 0.00 | 233.66 | 436.90 | 0.00 | 682.03 | 777.57 | low intensity |
|  | Acetazolamide | 9.76 | 5.01 | 4.74 | 0.03 | 26.81 | up | 742.83 | 2217.69 | 394.71 | 683.91 | 766.44 | 1214.63 | 0.00 | 1620.98 | 0.00 | 0.00 | 490.40 | 1407.73 | 794.94 |  |  |
|  | Rapid-Ripc | 9.41 | 3.30 | 6.11 | 0.01 | 68.87 | up | 1448.28 | 1413.59 | 1006.20 | 857.75 | 1291.44 | 42.12 | 1101.89 | 0.00 | 0.00 | 0.00 | 839.07 | 0.00 | 666.70 |  |  |
|  | Combined | 10.06 | 8.22 | 1.84 | 0.20 | 3.57 | up | 1212.04 | 824.70 | 1229.09 | 1636.74 | 1126.66 | 649.24 | 474.06 | 973.70 | 1252.14 | 1208.06 | 1012.78 | 0.00 | 966.60 |  |  |
| 6 | **ADAM12** | | | | | | | | | | | | | | | | | | | | | |
|  | Control | 8.69 | 1.31 | 7.38 | 0.00 | 166.52 | up | 469.09 | 875.70 | 494.70 | 109.09 | 467.81 | 467.16 | 0.00 | 230.49 | 0.00 | 0.00 | 0.00 | 0.00 | 259.50 | 401.18 | low intensity |
|  | Acetazolamide | 8.97 | 3.87 | 5.10 | 0.01 | 34.31 | up | 1561.12 | 441.99 | 246.54 | 193.64 | 791.98 | 611.24 | 0.00 | 275.03 | 0.00 | 0.00 | 379.40 | 93.53 | 382.87 |  |  |
|  | Rapid-Ripc | 7.91 | 2.54 | 5.37 | 0.02 | 41.33 | up | 540.85 | 308.11 | 1523.24 | 1020.90 | 740.10 | 0.00 | 93.50 | 0.00 | 0.00 | 0.00 | 409.49 | 0.00 | 386.35 |  |  |
|  | Combined | 9.21 | 6.20 | 3.01 | 0.09 | 8.07 | up | 1552.55 | 487.01 | 865.81 | 385.96 | 514.66 | 328.27 | 1188.42 | 756.38 | 426.76 | 0.00 | 405.90 | 0.00 | 575.98 |  |  |
| 7 | **JAM-B** |  |  |  |  |  |  |  |  |  |  |  |  |  |  |  |  |  |  |  |  |  |
|  | Control | 9.54 | 4.35 | 5.18 | 0.02 | 36.32 | up | 1097.73 | 629.77 | 880.54 | 510.26 | 620.82 | 858.97 | 341.77 | 558.02 | 0.00 | 0.00 | 379.03 | 0.00 | 489.74 | 510.80 | low intensity |
|  | Acetazolamide | 9.34 | 5.62 | 3.72 | 0.03 | 13.17 | up | 393.87 | 1651.45 | 463.18 | 523.08 | 682.37 | 682.07 | 0.00 | 693.71 | 186.12 | 0.00 | 406.01 | 267.21 | 495.76 |  |  |
|  | Rapid-Ripc | 9.24 | 2.96 | 6.27 | 0.01 | 77.37 | up | 745.67 | 712.01 | 543.69 | 581.82 | 811.19 | 351.95 | 375.62 | 0.00 | 0.00 | 0.00 | 598.60 | 0.00 | 393.38 |  |  |
|  | Combined | 9.68 | 7.58 | 2.10 | 0.14 | 4.30 | up | 804.92 | 721.15 | 727.30 | 688.54 | 864.70 | 1210.03 | 287.93 | 730.49 | 420.34 | 0.00 | 938.66 | 578.03 | 664.34 |  |  |
| 8 | **CD48** | | | | | | | | | | | | | | | | | | | | | |
|  | Control | 9.69 | 4.38 | 5.31 | 0.02 | 39.56 | up | 828.73 | 575.70 | 807.52 | 1247.80 | 769.66 | 852.71 | 0.00 | 262.91 | 0.00 | 897.79 | 348.84 | 0.00 | 549.31 | 493.49 | low intensity |
|  | Acetazolamide | 9.34 | 4.33 | 5.01 | 0.02 | 32.13 | up | 1258.11 | 719.65 | 545.59 | 578.13 | 581.03 | 442.56 | 0.00 | 547.95 | 0.00 | 0.00 | 395.72 | 308.39 | 448.09 |  |  |
|  | Rapid-Ripc | 9.20 | 4.22 | 4.99 | 0.01 | 31.73 | up | 1034.43 | 603.41 | 1244.67 | 541.67 | 798.00 | 123.58 | 270.97 | 0.00 | 357.97 | 0.00 | 421.76 | 0.00 | 449.71 |  |  |
|  | Combined | 9.14 | 7.23 | 1.92 | 0.14 | 3.77 | up | 922.28 | 762.67 | 708.04 | 444.84 | 531.74 | 273.61 | 300.48 | 1044.09 | 538.87 | 93.72 | 701.73 | 0.00 | 526.84 |  |  |
| 9 | **Lymphotactin** | | | | | | | | | | | | | | | | | | | | | |
|  | Control | 8.65 | 9.65 | -1.01 | 0.00 | 0.50 | down | 742.53 | 405.34 | 346.33 | 286.69 | 419.25 | 327.39 | 821.17 | 648.05 | 1200.08 | 603.97 | 861.24 | 816.41 | 623.21 | 669.87 | low intensity |
|  | Acetazolamide | 9.26 | 9.95 | -0.69 | 0.03 | 0.62 | down | 630.34 | 446.33 | 629.91 | 423.24 | 341.35 | 2047.79 | 837.10 | 771.05 | 1214.78 | 690.89 | 1118.06 | 1526.15 | 889.75 |  |  |
|  | Rapid-Ripc | 8.59 | 9.67 | -1.08 | 0.00 | 0.47 | down | 432.04 | 504.63 | 395.99 | 292.30 | 306.20 | 419.54 | 870.80 | 1026.30 | 655.15 | 839.65 | 999.94 | 600.90 | 611.95 |  |  |
|  | Combined | 8.29 | 9.57 | -1.28 | 0.00 | 0.41 | down | 648.99 | 205.94 | 338.56 | 361.93 | 205.53 | 275.81 | 1028.80 | 707.60 | 769.33 | 745.07 | 682.93 | 684.17 | 554.55 |  |  |
| 10 | **GRO** | | | | | | | | | | | | | | | | | | | | | |
|  | Control | 13.89 | 14.92 | -1.02 | 0.01 | 0.49 | down | 12737.16 | 12062.81 | 27571.66 | 31514.75 | 14308.95 | 6519.48 | 29653.23 | 22982.29 | 45868.19 | 28919.82 | 34382.13 | 28199.56 | 24560.00 | 27412.47 | ELISA kit not available |
|  | Acetazolamide | 14.33 | 15.11 | -0.78 | 0.09 | 0.58 | down | 14616.97 | 13029.27 | 31209.51 | 22506.53 | 13276.40 | 43422.98 | 51182.86 | 45427.58 | 65509.64 | 13984.57 | 29018.04 | 32251.79 | 31286.35 |  |  |
|  | Rapid-Ripc | 14.06 | 15.21 | -1.15 | 0.02 | 0.45 | down | 21365.40 | 19353.73 | 17959.27 | 11405.96 | 7729.30 | 37274.16 | 55922.30 | 28088.21 | 22371.82 | 22253.20 | 65223.53 | 58068.91 | 30584.65 |  |  |
|  | Combined | 13.71 | 14.87 | -1.16 | 0.01 | 0.45 | down | 6468.07 | 29384.37 | 15625.50 | 9735.67 | 13443.56 | 14600.37 | 40633.07 | 38924.94 | 16191.62 | 21510.58 | 39073.17 | 33035.60 | 23218.88 |  |  |
| 11 | **Tie-1** | | | | | | | | | | | | | | | | | | | | | |
|  | Control | 8.06 | 2.97 | 5.09 | 0.03 | 34.05 | up | 1770.61 | 246.96 | 2010.09 | 0.00 | 546.52 | 758.06 | 669.37 | 350.69 | 0.00 | 0.00 | 0.00 | 0.00 | 529.36 | 620.60 | low intensity |
|  | Acetazolamide | 7.52 | 6.24 | 1.28 | 0.20 | 2.43 | up | 1150.33 | 139.40 | 84.75 | 0.00 | 837.23 | 3333.43 | 603.00 | 231.65 | 0.00 | 0.00 | 644.15 | 2065.18 | 757.43 |  |  |
|  | Rapid-Ripc | 8.32 | 2.88 | 5.44 | 0.03 | 43.37 | up | 266.05 | 2142.13 | 1057.23 | 1265.91 | 1407.90 | 0.00 | 278.35 | 0.00 | 0.00 | 0.00 | 580.64 | 0.00 | 583.18 |  |  |
|  | Combined | 9.23 | 4.72 | 4.52 | 0.03 | 22.88 | up | 1380.33 | 241.03 | 858.13 | 215.57 | 1649.70 | 460.08 | 398.78 | 500.06 | 0.00 | 0.00 | 1645.63 | 0.00 | 612.44 |  |  |
| 12 | **PDGF-AB** | | | | | | | | | | | | | | | | | | | | | |
|  | Control | 11.30 | 12.86 | -1.57 | 0.00 | 0.34 | down | 960.50 | 755.31 | 6864.31 | 5236.32 | 1528.50 | 6303.48 | 8804.44 | 2333.25 | 19979.92 | 7185.22 | 4498.08 | 12781.37 | 6435.89 | 5697.99 | - |
|  | Acetazolamide | 12.33 | 12.60 | -0.26 | 0.32 | 0.83 | down | 8925.61 | 2403.01 | 10416.83 | 6873.56 | 4251.14 | 2901.73 | 11382.92 | 2598.43 | 9496.47 | 16164.18 | 2619.35 | 4790.92 | 6902.01 |  |  |
|  | Rapid-Ripc | 11.38 | 12.70 | -1.32 | 0.02 | 0.40 | down | 2586.24 | 3224.85 | 1584.31 | 3513.60 | 931.16 | 8291.81 | 2467.36 | 10453.55 | 11288.70 | 19052.32 | 1176.72 | 13545.69 | 6509.69 |  |  |
|  | Combined | 10.62 | 11.81 | -1.19 | 0.02 | 0.44 | down | 1111.40 | 3628.44 | 1234.25 | 1772.31 | 691.88 | 2453.27 | 4134.19 | 1663.09 | 3684.84 | 5934.03 | 2042.22 | 6982.36 | 2944.36 |  |  |
| 13 | **BMPR-1B** | | | | | | | | | | | | | | | | | | | | | |
|  | Control | 9.73 | 4.33 | 5.40 | 0.02 | 42.25 | up | 1180.58 | 588.57 | 1051.52 | 392.90 | 866.08 | 1489.35 | 0.00 | 399.81 | 0.00 | 0.00 | 378.76 | 430.35 | 564.83 | 653.84 | low intensity |
|  | Acetazolamide | 9.94 | 5.97 | 3.97 | 0.04 | 15.65 | up | 612.00 | 901.07 | 618.66 | 1230.38 | 1492.10 | 1404.97 | 0.00 | 627.19 | 150.02 | 0.00 | 1338.54 | 474.12 | 737.42 |  |  |
|  | Rapid-Ripc | 8.26 | 4.36 | 3.90 | 0.04 | 14.98 | up | 1222.70 | 509.21 | 2203.92 | 709.22 | 863.18 | 0.00 | 225.09 | 0.00 | 767.18 | 0.00 | 430.18 | 0.00 | 577.56 |  |  |
|  | Combined | 9.79 | 6.35 | 3.44 | 0.08 | 10.85 | up | 1961.40 | 629.81 | 744.47 | 899.08 | 744.13 | 764.26 | 1142.33 | 838.11 | 606.46 | 0.00 | 496.55 | 0.00 | 735.55 |  |  |
| 14 | **Siglec-10** | | | | | | | | | | | | | | | | | | | | | |
|  | Control | 9.77 | 4.37 | 5.40 | 0.01 | 42.08 | up | 1185.06 | 1638.33 | 737.84 | 660.15 | 650.30 | 705.22 | 370.28 | 808.45 | 0.00 | 0.00 | 260.77 | 0.00 | 584.70 | 540.63 | low intensity |
|  | Acetazolamide | 9.67 | 5.85 | 3.82 | 0.04 | 14.08 | up | 825.04 | 1067.55 | 735.44 | 642.48 | 1001.85 | 693.15 | 0.00 | 529.45 | 282.01 | 0.00 | 672.28 | 368.93 | 568.18 |  |  |
|  | Rapid-Ripc | 9.41 | 5.53 | 3.88 | 0.04 | 14.72 | up | 904.51 | 518.51 | 1148.73 | 801.86 | 443.91 | 516.28 | 321.50 | 0.00 | 509.80 | 0.00 | 277.89 | 212.59 | 471.30 |  |  |
|  | Combined | 9.35 | 7.07 | 2.28 | 0.11 | 4.85 | up | 696.61 | 663.47 | 1136.46 | 592.60 | 668.37 | 367.09 | 211.35 | 671.32 | 824.90 | 0.00 | 535.51 | 92.58 | 538.35 |  |  |

AveExp, average expression, calculated as average of log_2_(protein intensity+1); AveExp. post: average expression for post-hypoxia; AveExp.base, average expression for baseline; P Val, P value; FC, foldchange; Reg, regulation; S, subject; Ave.S, average intensity within each intervention group; Ave all, average intensity in all intervention groups. 1 Reference: Skjaerbaek C et al. Circadian variation in serum free and total insulin-like growth factor (IGF)-I and IGF-II in untreated and treated. *Clin Endocrinol (Oxf)*.2000;52(1):25-33. 2 Intensity greater than 1500 is considered suitable for further validation.


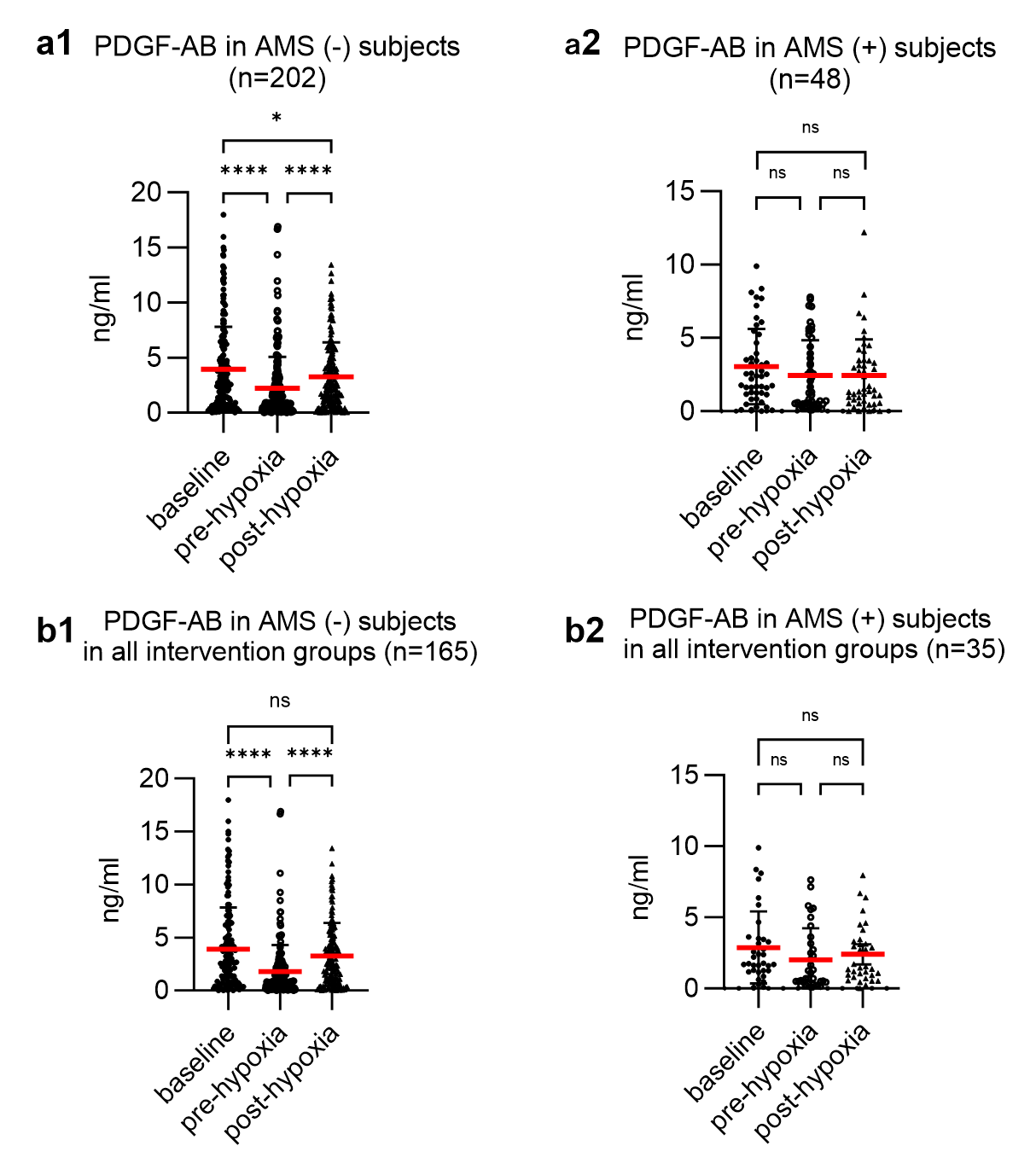


Figure S3. Analysis of PDGF-AB levels in AMS (+) and AMS (-) subjects. (a) Analysis of PDGF-AB levels in all subjects (n=250) based on AMS (+)/(-) subgroups. A significant reduction in PDGF-AB levels from baseline to pre-hypoxia was observed in AMS (-) subjects (a1), but not in AMS (+) subjects (a2). The reduction of PDGF-AB levels was significantly different between the two groups (*P* = 0.043). (b) Analysis of PDGF-AB levels in subjects of the intervention groups (n=200) based on AMS (+)/(-) subgroups. A similar significant reduction in PDGF-AB levels was observed in AMS (-) subjects (b1), but not in AMS (+) subjects (b2). Data are individual values, and mean with SD are showed. Ns: non-significant; ^*^*P* < 0.05, ^****^*P* < 0.0001.

**
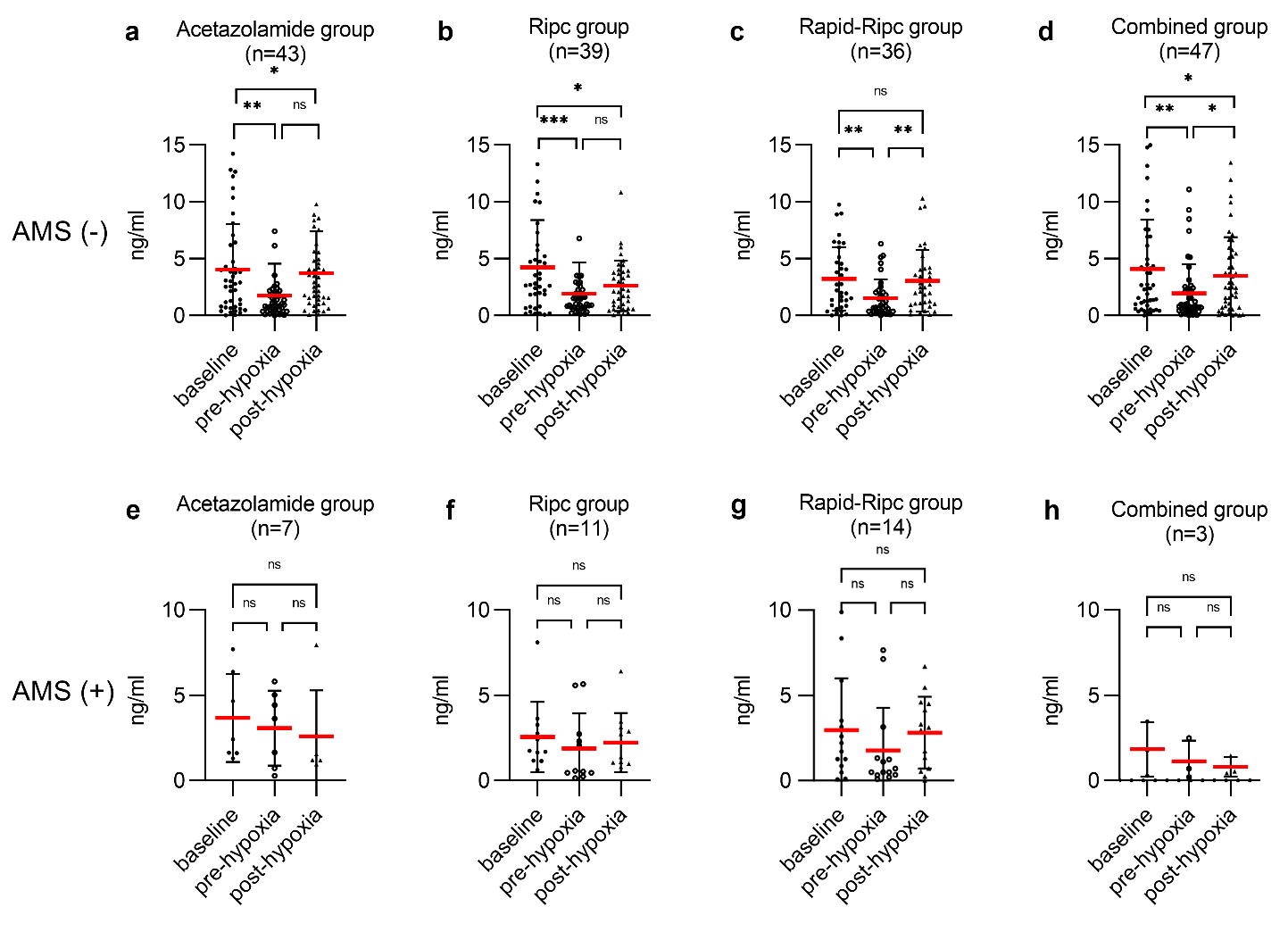
**Figure S4. Analysis of PDGF-AB levels among different intervention groups based on AMS (-)/(+) subgroups. In AMS (-) subjects, Acetazolamide (a), Ripc (b), and Rapid-Ripc (c) all reduced PDGF-AB levels from baseline to pre-hypoxia, and after the combination of acetazolamide and Rapid-Ripc (Combined group), the reduction was still significant (d). While in AMS (+) subjects, there was no significant decrease in PDGF-AB levels in either of the groups (e-h). Data are individual values, and mean with SD are showed. Ns: non-significant; **P* < 0.05, ***P* < 0.01, ****P* < 0.001.

#
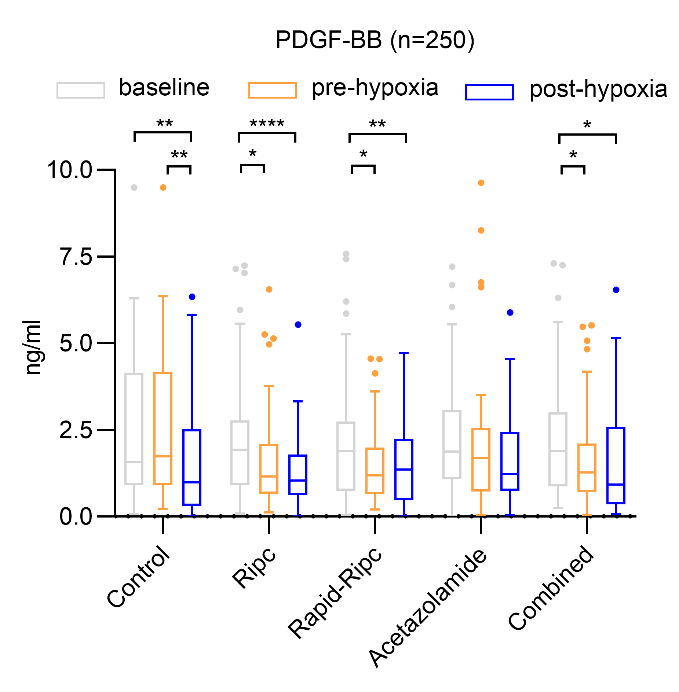


Figure S5. PDGF-BB levels in all subjects validated by ELISA. PDGF-BB levels at baseline, pre-, and post-hypoxia in each group are presented. A significant reduction of PDGF-BB levels was observed between baseline and pre-hypoxia in Ripc, Rapid-Ripc, and Combined group, but not in Acetazolamide group (baseline to pre-hypoxia, mean difference: Control group: 0.09 ng/ml, 95% CI: -0.1 to 0.3, *P* = 0.448; Ripc group: -0.66 ng/ml, 95% CI:-1.3 to -0.0, *P* = 0.036; Rapid-Ripc group: -0.75 ng/ml, 95% CI: -1.4 to -0.1, *P* = 0.020; Acetazolamide group: -0.14 ng/ml, 95% CI: -0.8 to 0.6, *P* = 0.875; Combined group: -0.60 ng/ml, -1.2 to -0.1, *P* = 0.022). The pre-hypoxic PDGF-BB levels in Rapid-Ripc were significantly lower than those in Control group (*P* = 0.032). Data are presented in box-whisker plots using Tukey method.

**
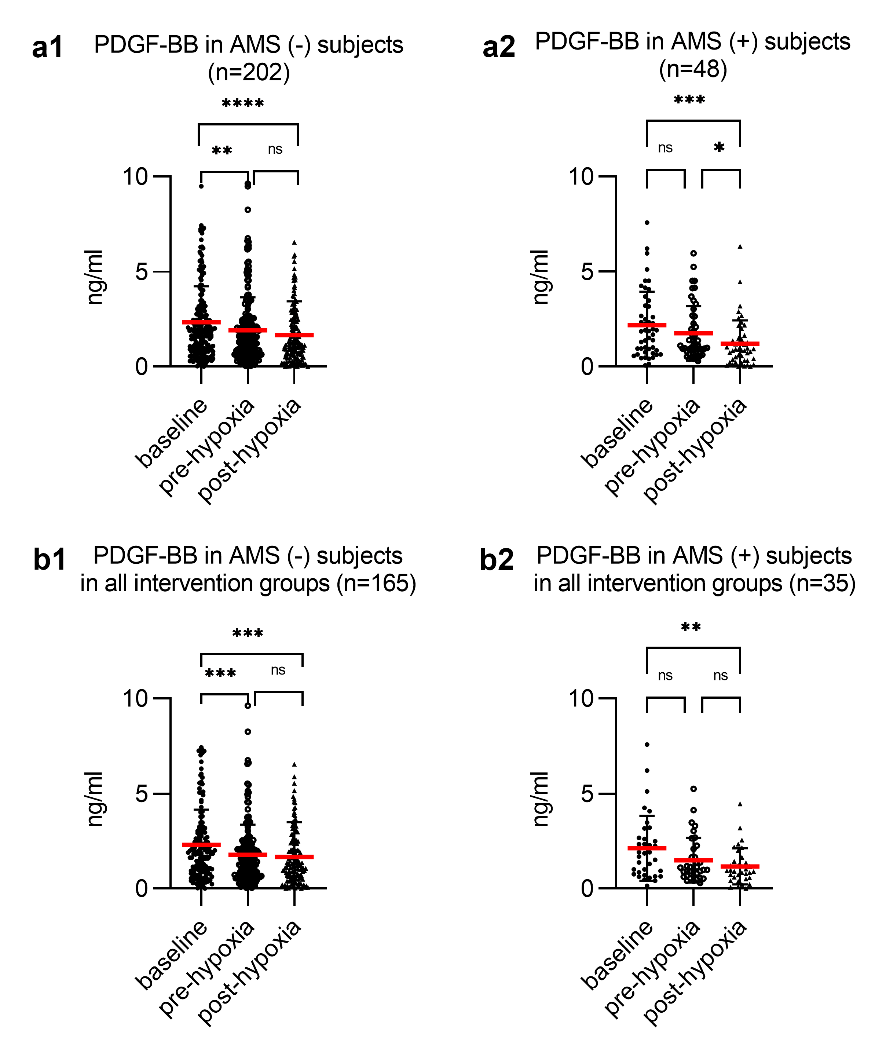
**

Figure S6. Analysis of PDGF-BB levels in AMS (+) and AMS (-) subjects. (a) Analysis of PDGF-BB levels in all subjects (n=250) based on AMS (+)/(-) subgroups. A significant reduction of PDGF-BB levels from baseline to pre-hypoxia was observed in AMS (-) subjects (a1; -0.42 ng/ml, 95% CI: -0.7 to -0.1, *P* = 0.002), but not in AMS (+) subjects (a2; -0.44 ng/ml, 95% CI: -1.0 to 0.1, *P* = 0.174). The reduction of PDGF-BB levels was not statistically different between AMS (+) and AMS (-) groups (-0.42 ng/ml vs -0.44 ng/ml, 95%CI: -0.55 to 0.52, *P* = 0.946). (b) Analysis of PDGF-BB levels subjects of the intervention groups (n=200) based on AMS (+)/(-) subgroups. A similar significant reduction in PDGF-BB levels was observed in AMS (-) subjects (b1; -0.53 ng/ml, 95% CI: -0.9 to -0.2, *P* < 0.001), but not in AMS (+) subjects (b2; -0.64 ng/ml, 95% CI: -1.4 to 0.2, *P* = 0.133). Data are individual values, and mean with SD are showed. Ns: non-significant; **P* < 0.05, ***P* < 0.01, ****P* < 0.001, *****P* <0.0001.


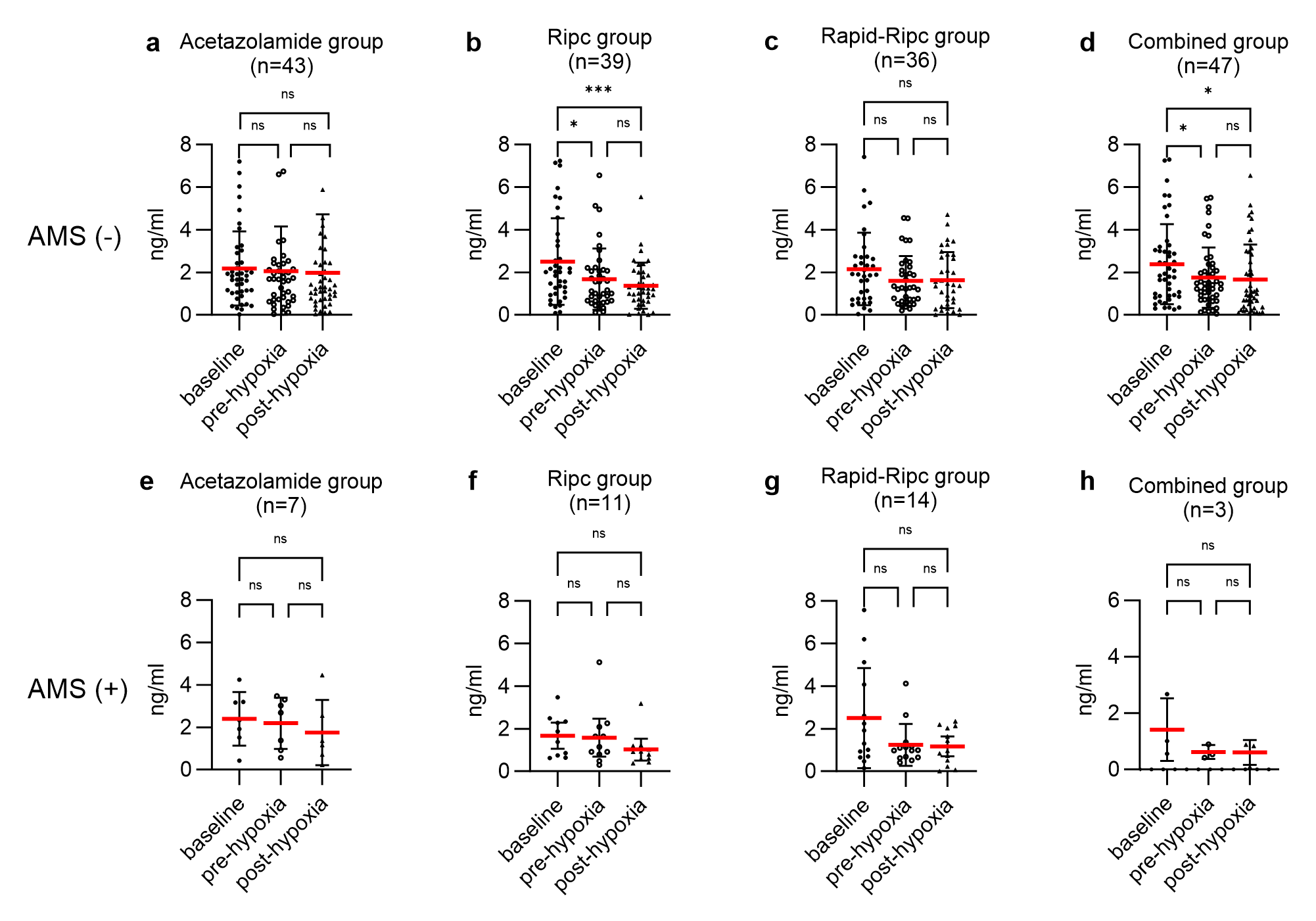
Figure S7. Analysis of PDGF-BB levels among different intervention groups based on AMS (-)/(+) subgroups. Ripc significantly reduced PDGF-BB levels from baseline to pre-hypoxia in AMS (-) subjects (b; -0.83 ng/ml, 95% CI: -1.6 to -0.1, *P* = 0.023), but did not in AMS (+) subjects (f; -0.10 ng/ml, 95% CI: -1.5 to 1.3, *P* = 0.980). Rapid-Ripc failed to show this effect (c: -0.56 ng/ml, 95% CI: -1.2 to 0.1, *P* = 0.121; g: -1.25 ng/ml, 95% CI: -3.0 to 0.5, *P* = 0.173), but Combined group was effective, with a significant decrease in PDGF-BB levels in AMS (-) (d: -0.63 ng/ml, 95% CI: -1.2 to -0.0, *P* = 0.036). Acetazolamide did not show the decrease in PDGF-BB levels either in AMS (-) subjects or in AMS (+) subjects (a: -0.13 ng/ml, 95% CI: -0.9 to 0.7, *P* = 0.918, e: -0.21 ng/ml, 95% CI: -1.4 to 0.9, *P* = 0.848). Data are individual values, and mean with SD are showed. Ns: non-significant; **P* < 0.05, ****P* < 0.001.

# Table S8. Associations analyses between SNPs and AMS.

| Model | Allele/Genotype | AMS (+)  n=42 | AMS (-)  n=191 | OR (95% CI) | *P* value |
| --- | --- | --- | --- | --- | --- |
| ***PDGFA* rs62433334 G>C** | | | | | |
| Allele | G | 44 (52.4%) | 196 (51.0%) | - | 0.82 |
|  | C | 40 (47.6%) | 188 (49.0%) | - |  |
| Codominant | GG | 14 (33.3%) | 50 (26.0%) | 1 | 0.37 |
|  | CG | 16 (38.1%) | 96 (50.0%) | 1.68 (0.76-3.72) |  |
|  | CC | 12 (28.6%) | 46 (24.0%) | 1.07 (0.45-2.56) |  |
| Dominant | GG | 14 (33.3%) | 50 (26.0%) | 1 | 0.34 |
|  | CG/CC | 28 (66.7%) | 142 (74.0%) | 1.42 (0.69-2.91) |  |
| Recessive | GG/CG | 30 (71.4%) | 146 (76.0%) | 1 | 0.54 |
|  | CC | 12 (28.6%) | 46 (24.0%) | 0.79 (0.37-1.66) |  |
| Log-additive | - | - | - | 1.05 (0.66-1.67) | 0.83 |
| ***PDGFB* rs1800817 T>G** | | | | | |
| Allele | T | 77 (89.5%) | 349 (90.9%) | - | 0.70 |
|  | G | 9 (10.5%) | 35 (9.1%) | - |  |
| Codominant | TT | 35 (81.4%) | 160 (83.3%) | 1 | 0.92 |
|  | TG | 7 (16.3%) | 29 (15.1%) | 0.91 (0.37-2.24) |  |
|  | GG | 1 (2.3%) | 3 (1.6%) | 0.66 (0.07-6.50) |  |
| Dominant | TT | 35 (81.4%) | 160 (83.3%) | 1 | 0.76 |
|  | TG/GG | 8 (18.6%) | 32 (16.7%) | 0.87 (0.37-2.06) |  |
| Recessive | TT/TG | 42 (97.7%) | 189 (98.4%) | 1 | 0.74 |
|  | GG | 1 (2.3%) | 3 (1.6%) | 0.67 (0.07-6.57) |  |
| Log-additive | - | - | - | 0.87 (0.42-1.82) | 0.71 |
| ***PDGFB* rs1800818 T>C** | | | | | |
| Allele | T | 76 (90.5%) | 347 (90.8%) | - | 0.92 |
|  | C | 8 (9.5%) | 35 (9.2%) | - |  |
| Codominant | TT | 35 (83.3%) | 159 (83.2%) | 1 | 0.93 |
|  | TC | 6 (14.3%) | 29 (15.2%) | 1.06 (0.41-2.76) |  |
|  | CC | 1 (2.4%) | 3 (1.6%) | 0.66 (0.07-6.54) |  |
| Dominant | TT | 35 (83.3%) | 159 (83.2%) | 1 | 0.99 |
|  | TC/CC | 7 (16.7%) | 32 (16.8%) | 1.01 (0.41-2.47) |  |
| Recessive | TT/TC | 41 (97.6%) | 188 (98.4%) | 1 | 0.73 |
|  | CC | 1 (2.4%) | 3 (1.6%) | 0.65 (0.06-6.45) |  |
| Log-additive | - | - | - |  |  |
| ***PDGFB* rs2285099 C>T** | | | | | |
| Allele | C | 68 (81.0%) | 308 (80.6%) | - | 0.946 |
|  | T | 16 (19.0%) | 74 (19.4%) | - |  |
| Codominant | CC | 27 (64.3%) | 123 (64.4%) | 1 | 0.96 |
|  | TC | 14 (33.3%) | 62 (32.5%) | 0.97 (0.48-1.99) |  |
|  | TT | 1 (2.4%) | 6 (3.1%) | 1.32 (0.15-11.39) |  |
| Dominant | CC | 27 (64.3%) | 123 (64.4%) | 1 | 0.99 |
|  | TC/TT | 15 (35.7%) | 68 (35.6%) | 1.00 (0.50-2.00) |  |
| Recessive | CC/TC | 41 (97.6%) | 185 (96.6%) | 1 | 0.79 |
|  | TT | 1 (2.4%) | 6 (3.1%) | 1.33 (0.16-11.35) |  |
| Log-additive | - | - | - | 1.02 (0.55-1.89) | 0.94 |
| ***PDGFB* rs2285094 T>C** | | | | |  |
| Allele | T | 69 (82.1%) | 309 (80.9%) | - | 0.79 |
|  | C | 15 (17.9%) | 73 (19.1%) | - |  |
| Codominant | TT | 28 (66.7%) | 124 (64.9%) | 1 | 0.95 |
|  | TC | 13 (30.9%) | 61 (31.9%) | 1.06 (0.51-2.19) |  |
|  | CC | 1 (2.4%) | 6 (3.1%) | 1.35 (0.16-11.71) |  |
| Dominant | TT | 28 (66.7%) | 124 (64.9%) | 1 | 0.83 |
|  | TC/CC | 14 (33.3%) | 67 (35.1%) | 1.08 (0.53-2.19) |  |
| Recessive | TT/TC | 41 (97.6%) | 185 (96.9%) | 1 | 0.79 |
|  | CC | 1 (2.4%) | 6 (3.1%) | 1.33 (0.16-11.35) |  |
| Log-additive | - | - | - | 1.09 (0.58-2.04) | 0.79 |

# Table S9. Decrease in PDGF-AB levels from baseline to pre-hypoxia in different SNP subgroups.

| Subgroups | Decrease in PDGF-AB levels from baseline to pre-hypoxia (ng/ml) (mean with 95% CI) | *P* value |
| --- | --- | --- |
| **rs2070958** |  |  |
| Acetazolamide TT (n=10) | -2.12 (-3.87 to -0.36) | 0.024 |
| Acetazolamide TC (n=24) | -3.36 (-5.70 to -1.01) | 0.0044 |
| Acetazolamide CC (n=12) | -1.84 (-4.31 to 0.63) | 0.16 |
| Ripc TT (n=5) | -3.70 (-7.00 to -0.41) | 0.020 |
| Ripc TC (n=30) | -2.34 (-3.98 to -0.70) | 0.0040 |
| Ripc CC (n=11) | -0.48 (-2.59 to 1.64) | 0.81 |
| Rapid-Ripc TT (n=15) | -0.85 (-2.48 to 0.79) | 0.39 |
| Rapid-Ripc TC (n=16) | -2.34 (-4.12 to 0.66) | 0.0071 |
| Rapid-Ripc CC (n=15) | -1.29 (-4.33 to 1.76) | 0.53 |
| **rs9690350** |  |  |
| Acetazolamide CC (n=6) | -1.73 (-4.17 to 0.71) | 0.15 |
| Acetazolamide CG (n=22) | -2.78 (-5.00 to -0.54) | 0.013 |
| Acetazolamide GG (n=18) | -2.91 (-5.37 to -0.45) | 0.019 |
| Ripc CC (n=5) | -3.70 (-7.00 to -0.41) | 0.036 |
| Ripc CG (n=30) | -2.75 (-4.38 to -1.13) | 0.0007 |
| Ripc GG (n=11) | -0.71 (-3.44 to 2.01) | 0.76 |
| Rapid-Ripc CC (n=13) | -1.09 (-2.91 to 0.74) | 0.29 |
| Rapid-Ripc CG (n=12) | -2.78 (-5.12 to -0.44) | 0.021 |
| Rapid-Ripc GG (n=22) | -1.29 (-3.37 to 0.79) | 0.28 |
| **rs1800814** |  |  |
| Acetazolamide GG (n=13) | -1.63 (-3.94 to 0.69) | 0.20 |
| Acetazolamide GA (n=24) | -3.42 (-5.73 to -1.1) | 0.0034 |
| Acetazolamide AA (n=9) | -2.29 (-4.24 to -0.33) | 0.027 |
| Ripc GG (n=11) | -0.48 (-2.59 to 1.63) | 0.81 |
| Ripc GA (n=30) | -2.84 (-4.53 to -1.14) | 0.0008 |
| Ripc AA (n=5) | -3.70 (-7.00 to -0.41) | 0.036 |
| Rapid-Ripc GG (n=14) | -1.03 (-4.25 to 2.13) | 0.68 |
| Rapid-Ripc GA (n=18) | -2.28 (-3.96 to -0.60) | 0.0076 |
| Rapid-Ripc (n=14) | -1.05 (-2.73 to 0.62) | 0.26 |
